# Testosterone supplementation improves insulin responsiveness in HFD fed male T2DM mice and potentiates insulin signaling in the skeletal muscle and C2C12 myocyte cell line

**DOI:** 10.1371/journal.pone.0224162

**Published:** 2019-11-06

**Authors:** Madhuraka Pal, Jasim Khan, Ravi Kumar, Avadhesha Surolia, Sarika Gupta

**Affiliations:** 1 Molecular Science Laboratory, National Institute of Immunology, New Delhi, India; 2 Molecular Toxicology Laboratory, Department of Medical Elementology and Toxicology, School of Chemical and Life Sciences, Jamia Hamdard, New Delhi, India; 3 Molecular Biophysics Unit, Indian Institute of Science, Bengaluru, Karnataka, India; Tohoku University, JAPAN

## Abstract

**Background:**

Type 2 Diabetes Mellitus (T2DM) is characterised by hyperglycemia due to the incidence of insulin resistance. Testosterone supplementation has been shown to have a positive co-relation with improved glycemic control in T2DM males. Clinical studies have reported that Androgen Replacement Therapy (ART) to hypogonadic males with T2DM resulted in improved glycemic control and metabolic parameters, but, these studies did not address in detail how testosterone acted on the key glucose homeostatic organs.

**Method:**

In this study, we delineate the effect of testosterone supplementation to high-fat diet (HFD) induced T2DM in male C57BL6J mice and the effect of testosterone supplementation on the skeletal muscle insulin responsiveness. We also studied the effect of testosterone on the insulin signaling pathway proteins in C2C12 myocyte cells to validate the in vivo findings.

**Results:**

We found that testosterone had a potentiating effect on the skeletal muscle insulin signaling pathway to improve glycaemic control. We demonstrate that, in males, testosterone improves skeletal muscle insulin responsiveness by potentiating the PI3K-AKT pathway. The testosterone treated animals showed significant increase in the skeletal muscle Insulin Receptor (IR), p85 subunit of PI3K, P-GSK3α (Ser-21), and P-AKT (Ser-473) levels as compared to the control animals; but there was no significant change in total AKT and GSK3α. Testosterone supplementation inhibited GSK3α in the myocytes in a PI3K/AKT pathway dependent manner; on the other hand GSK3β gene expression was reduced in the skeletal muscle upon testosterone supplementation.

**Conclusion:**

Testosterone increases insulin responsiveness by potentiating insulin signaling in the skeletal muscle cells, which is in contrast to the increased insulin resistance in the liver of testosterone treated T2DM male animals.

## Introduction

Type 2 Diabetes Mellitus (T2DM), also known as Non-Insulin Dependent Diabetes Mellitus, one of a components of Metabolic Syndrome (Met S) and has a very complex pathology. It is one of the major reasons of morbidity and mortality across the world. Insulin resistance, a pathological condition characterised by the body’s inability to effectively utilise insulin produced by the β-islets of pancreas, results in hyperglycemia in T2DM. It is a condition that is a precursor to developing type 2 diabetes and marked by the incidence of hyperinsulinemia. One of the key treatment strategies of T2DM is to increase insulin sensitivity of the tissues that take up glucose in response to insulin in the body [1, 2].

A number of physiological factors are related to the propagation and pathophysiology of T2DM. Testosterone supplementation has been shown to have a positive co-relation with improved glycemic control and increased insulin responsiveness in hypogonadic males. Androgen Replacement Therapy (ART) in hypogonadic males or males with sub-normal testosterone, who also had T2DM, showed improvement in their glucose homeostasis parameters like reduced fasting and post prandial blood glucose levels (BGL) along with reduction in waist circumference and lower levels of triglycerides [3, 4, 5]. Studies in isolated rat myocytes have shown that testosterone treatment increase the insulin responsive glucose transporter, Glut 4 translocation to plasma membrane of the skeletal muscle, resulting in increased glucose uptake by skeletal muscle cells [6]. Glut 4 translocation can be both insulin dependent and independent as certain bioactive molecules can increase Glut 4 translocation in skeletal muscle and testosterone activates Glut 4 translocation in 3T3-L1 adipocytes by stimulating the LKB1/AMPK pathway, in an insulin independent manner [7, 8]. However, in these studies, the underlying mechanism of action of testosterone on the insulin signaling pathway and insulin responsiveness in the major glucose homeostatic tissues, resulting in the enhanced glycemic control remained unanswered. In our study, we address how testosterone acted on the key glucose homeostatic organs, liver and skeletal muscle, to bring about this outcome. In a previous study [9] we observed that the control or vehicle treated T2DM male mice and testosterone treated T2DM male mice did not show any significant change (p-value> 0.1) in the serum levels of the key glucose homeostatic hormones and cytokines like insulin, glucagon, C-peptide, leptin, GLP-1 Il-6, MCP-1 and TNFα; and thus, the testosterone mediated improvement in glycemic control in T2DM male mice is due to the alteration in signaling pathways in the tissues involved in glycemic control. In a previous study we demonstrated that the FOXO1-Androgen Receptor interaction in the liver of the testosterone treated male T2DM mice inhibited the gluconeogenic action of FOXO1, decreasing the PEPCK levels, thereby reducing fasting BGL, despite an increased hepatic insulin resistance [9]. In this study, we demonstrate that testosterone supplementation improves insulin responsiveness by potentiating insulin signaling in skeletal muscle of HFD induced T2DM male mice.

## Materials and methods

### Animal experiments

Animal models were generated as described before [9] with approval of the animal care and ethics committee of National Institute of Immunology (IAEC No. 296/12). Briefly, 8 weeks old male C57BL6J mice were fed with 60% kcal fat diet or High Fat Diet (HFD) (from Research Diets. Inc. New Brunswick, NJ, USA, Cat. No. D12492) till the end of the experiment. Animals were randomly grouped into the treated and the control groups, after confirmation of T2DM model (see supplementary material). In treated group, 18 weeks age onwards till the end of the experiment, 8mg/kg body weight Testosterone Propionate (from Sigma Aldrich, St Louis, MO, USA) suspended in sesame oil was subcutaneously injected twice a week and the control group was treated with vehicle sesame oil. All experiments were performed after 24 hr of testosterone propionate/ vehicle treatment. Animals were randomly selected from the treated and the control groups for experiments with blinding.

For Glucose Tolerance test, the animals were fasted for 16 hours and 3mg/kg body weight D-Glucose (from Sigma Aldrich) was injected intraperitoneally and blood glucose level (BGL) measured every 30min for next 120 minutes. For Insulin Tolerance test, the animals were fasted for 2 hours and 0.75 IU insulin/kg body weight was injected intraperitoneally. Then BGL were measured every 30min for next 120 minutes. BGL was measured with the help of a glucometer (Roche) by taking a drop of blood from the tail vein.

For tissue isolation and lysate preparation, animals were euthanized by cervical dislocation, after 32 weeks of treatment, at the age of 50 weeks, skeletal muscle from hind limb removed completely, snap frozen and homogenised in chilled condition. The clear lysate was used for immunoblot and immuno precipitation studies. Animals were not fasted prior to sacrifice. In animals given insulin treatment, 0.75IU insulin/kg body weight was intraperitoneally injected without fasting and were euthanized after 1hr.

### Immuno blotting

For immuno blotting, equal amounts of protein (50μg) for each group were resolved by 10% or 12% SDS‐PAGE and then transferred onto PVDF membranes (Mdi, Membrane Technologies, Ambala, India). The membranes were then incubated with the appropriate primary antibody as indicated, overnight at 4°C. Bound antibodies were visualized using HRP-conjugated secondary antibodies. Primary antibodies were procured from Cell Signaling Technologies (Danvers, MA, USA) and Santa Cruz (Santa Cruz, CA, USA) (see supplementary material). HRP-conjugated secondary antibodies were procured from Santa Cruz (Santa Cruz, CA, USA). Blots were developed and images captured in Fuji LAS 3000 and densitometric analysis was done by normalising the blots with loading control—GAPDH or α-Tubulin.

### Plasma isolation and testosterone ELISA

Animals were bled at the age of 50 weeks after 24 hours of treatment. Testosterone levels in plasma were measured using Testosterone ELISA kit procured from DRG International, Germany (Catalogue No. EIA-1559). The experiment analyses were done as per the manufacturer’s protocol.

### *Ex-vivo* [^3^H] 2-DG uptake assay

Skeletal muscles were isolated from the hind limb of the treated and the control animals after 28 weeks of treatment. 1g tissue was weighed and washed in PBS. Each muscle was then incubated in 2 mL Krebs Henseleit buffer [KHB] supplemented with 0.1% bovine serum albumin [BSA] [KHB-BSA], 1 mmol/L 2-DG and 1μCi [^3^H]-2-DG (Perkin Elmer) for 30 min at 37°C. The muscle were then washed in PBS and lysed in 1% SDS. Scintillation fluid was added to the lysate and reading was taken in β-counter (Perkin Elmer). Assay method was adapted based on method described by Mitsuhashi et al., 2016 [8].

### Microarray analysis

For microarray analysis, skeletal muscle were isolated from 3 animals of each group (the treated and the control and age matched normal chow fed male C57BL6J mice) and was pooled. The RNA preparation from the pooled samples and microarray analysis was outsourced to Sandor Life Sciences Pvt. Ltd.

The analysis was performed by uploading the data onto Genome-studio software and analysis was performed considering normalization and FDR (False discovery rate). Filtering of the data was done based on detection p-value (for both case and control samples) and diff score / diff p-val (only for case).* Detection and Diff P-value cut off was taken as ≤ 0.05 (diff score cut off considered is ± 13). The genes and probes falling in these cut-off regions were considered as significant ones. From these results, diff score; diff p-val ≤- 13; 0.05 are down-regulated genes and diff score; diff p-val >+ 13; 0.05 are considered as up-regulated genes. From significant differential results, log2 ratio and fold change was calculated. Then further filtering was done based on fold change cut-offs and significant results were considered. * Detection p-value is indication of good quality signal and diff p-val is for differential analysis results.

### *In vitro* experiments

C2C12 cells (from ATCC), were grown in high glucose DMEM with 10% Horse serum and 1% anti-biotic anti-mycotic (all from GIBCO, Auckland, New Zealand) till 80% confluency. Cells were serum starved in serum free media for 6 hours before the experiment. Insulin, testosterone and LY294002 were procured from Sigma Aldrich. Cell lysates were used for Immunoblot.

Cells tested negative for mycoplasma contamination (EZ-PCR mycoplasma test kit, Biological Industries, Beit-Haemek, Israel was used).

### Glycogen content assay

Glycogen content was measured according to the spectrophotometric method of Roelandt et al., 2010 [10] (see supplementary material).

## Statistical analysis

The data have shown normal distribution. All values were presented as the mean and ±S.D. Statistical significance was estimated either by unpaired, two tailed Student’s *t*-test (for two comparisons) or two-way repeated measures ANOVA test (for more than two comparisons) followed by Bonferroni post hoc analysis. *P*-values less than 0.05 were considered significant.

## Results and discussion

### Increased glucose dispersal and insulin responsiveness in treated animals without any significant change in the body mass as compared to control ones

**Fig 1:**
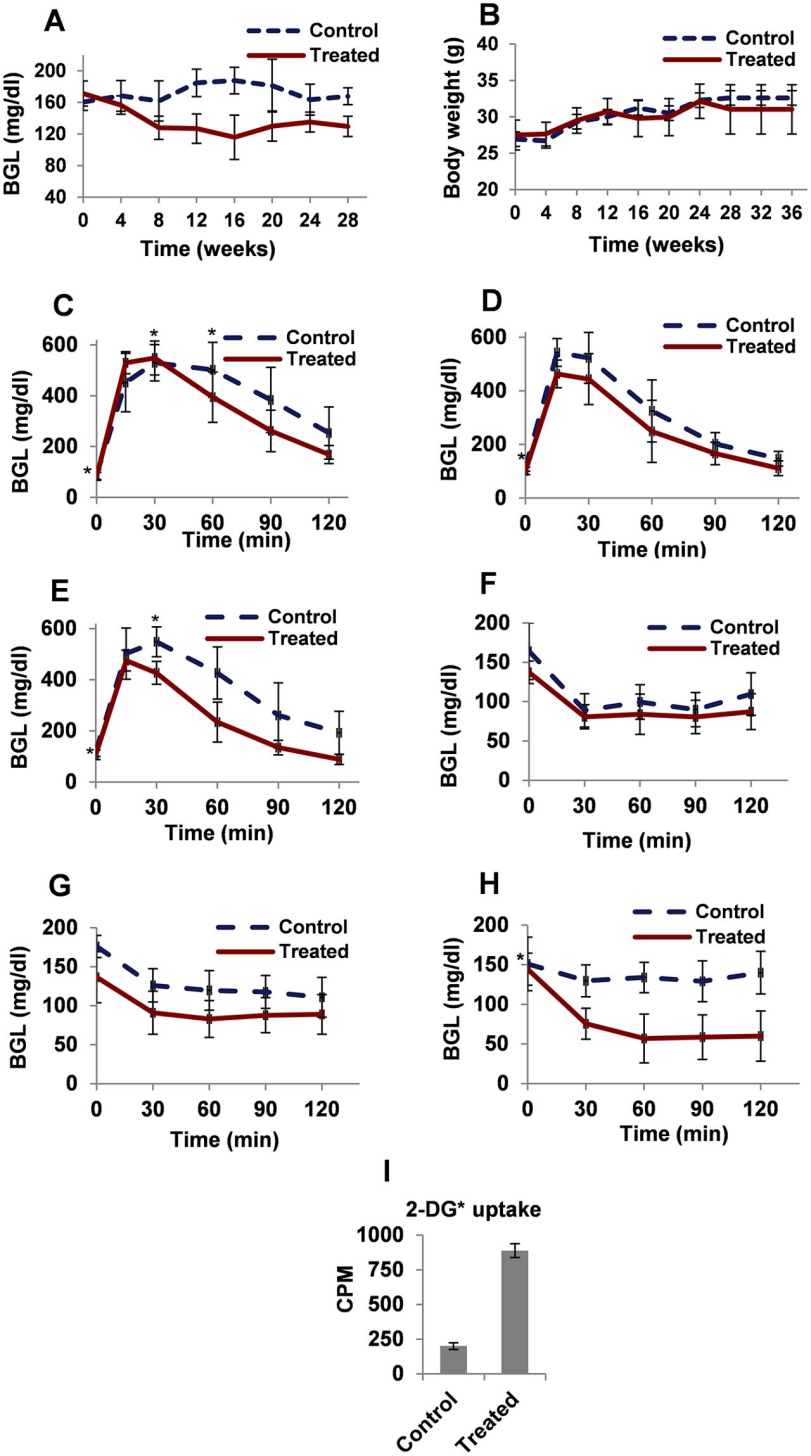
Increased glucose dispersal and insulin sensitivity in treated animals as compared to control ones without any significant change in the body weight. Fig 1A: PP BGL of the control and the treated animals. The data were analyzed by t-test, data represents mean ±S.D. n = 8, N = 2, p< 0.05. B: Body weight of the control and treated animals. The data were analyzed by t-test, data represents mean ±S.D. n = 8, N = 2, p> 0.1. Figs C-E: GTT of the control and the treated animals after 4 (Fig 1C), 16 (Fig 1D) and 32 (Fig 1E) weeks of treatment. Figs 1F-H: ITT of the control and the treated animals after 4 (Fig 1F), 16 (Fig 1G) and 32 (Fig 1H) weeks of treatment. The data were analyzed by t-test, data represents mean ±S.D. n = 8, N = 2, p< 0.05, * = p> 0.05. Fig I: *Ex-vivo* 2-DG* uptake in skeletal muscle of control and treated animals after 32 weeks of treatment. The data were analyzed by t-test, data represent mean ±S.D. of 3 independent experiments (n = 3), p< 0.05. J: Glycogen content assay in skeletal muscle of control and treated animals after 32 weeks of treatment. The data were analyzed by t-test, data represent mean ±S.D. of 3 independent experiments (n = 3), p< 0.05.

The glucose level in the blood rises after a carbohydrate rich meal and the excess glucose is taken up by body cells in response to the glucose induced insulin secretion. Post-prandial (PP) BGL is a marker of glucose dispersal in the body [11, 12]. In our study we observed that the treated animals had lower PP BGL compared to the control, throughout the experimental period of 28 weeks (Fig 1A), which indicated better glucose dispersal upon testosterone treatment.

The treated and the control animals showed no significant change in the body mass (p>0.1) over a period of 36 weeks (Fig 1B). The testosterone treatment to HFD fed male mice decreased the fat content over the period of treatment (<5% of total body mass) as compared to that in the control animals (>10% of body mass). The anabolic steroid, testosterone is known to increase the skeletal muscle mass and decrease adipose tissue mass [13] which might have resulted in no significant alteration in the body weight of the treated animals as compared to the control ones in our study. In our preliminary experiments, we observed that the myotubes were better formed in the skeletal muscle of the treated animals as compared to the control ones (data not shown).

To further check the efficacy of glucose dispersal in the animals, we performed Glucose Tolerance Test (GTT). The treated animals were more glucose tolerant as compared to the control ones (Fig 1C–1E). The insulin responsiveness or sensitivity is studied by performing Insulin Tolerance Test (ITT). The insulin responsive tissues take up glucose from blood in response to insulin, lowering the BGL. Individuals with T2DM do not efficiently utilise the administered insulin to lower the BGL [14, 15]. In the ITT, the treated animals were more insulin responsive as compared to the control ones (Fig 1F–1H). We also observed that glucose tolerance and insulin sensitivity in the treated animals improved with duration of treatment.

The plasma testosterone level in the control and the treated group were measured. The mean plasma testosterone level of the control mice was 5.11 ng/ml (±3.32) and was significantly less than that of the treated ones, which was 13.13 ng/ml (±5.74) (n = 10, p<0.005). The plasma testosterone level of the control animals were in the range described by Nelson et al., 1975 for age matched normal male C57BL6J mice (0.19–12.18 ng/ml) [16]. We found that the HFD fed animals were not hypogonadic and testosterone supplementation increases serum level to near normal range.

### Increased *ex vivo* glucose uptake and glycogen content in skeletal muscle of the treated animals

Skeletal muscle is one of the main organs that takes up excess glucose from the blood and stores as glycogen. The observed improved glycemic control in treated animals, prompted us to validate whether testosterone treatment promoted glucose uptake by the skeletal muscle and increase glycogen content. *Ex vivo* radioactive 2-deoxy glucose (2-DG) uptake assay was performed in isolated skeletal muscle of treated and control animals. We found that the treated animals showed increased radioactive 2-DG uptake as compared to control animals (Fig 1I). The glycogen content in the isolated skeletal muscle of the treated animals was significantly higher than that of the control ones (Fig 1J).

In our previous study [9], we found that the treated and control animals showed no significant alteration in intrinsic insulin and other glucose homeostatic hormone levels, but both groups showed significant alteration in intrinsic glucose homeostatic hormones and cytokine levels as compared to age matched normal chow fed C57BL6J male mice (S1 Table). Thus, this enhanced glycemic control in the treated animals could be due to altered insulin sensitivity in the skeletal muscle. Hence, insulin signalingin the skeletal muscle of the treated and control animals was examined.

### Increased insulin responsiveness in skeletal muscle of treated animals

**Fig 2:**
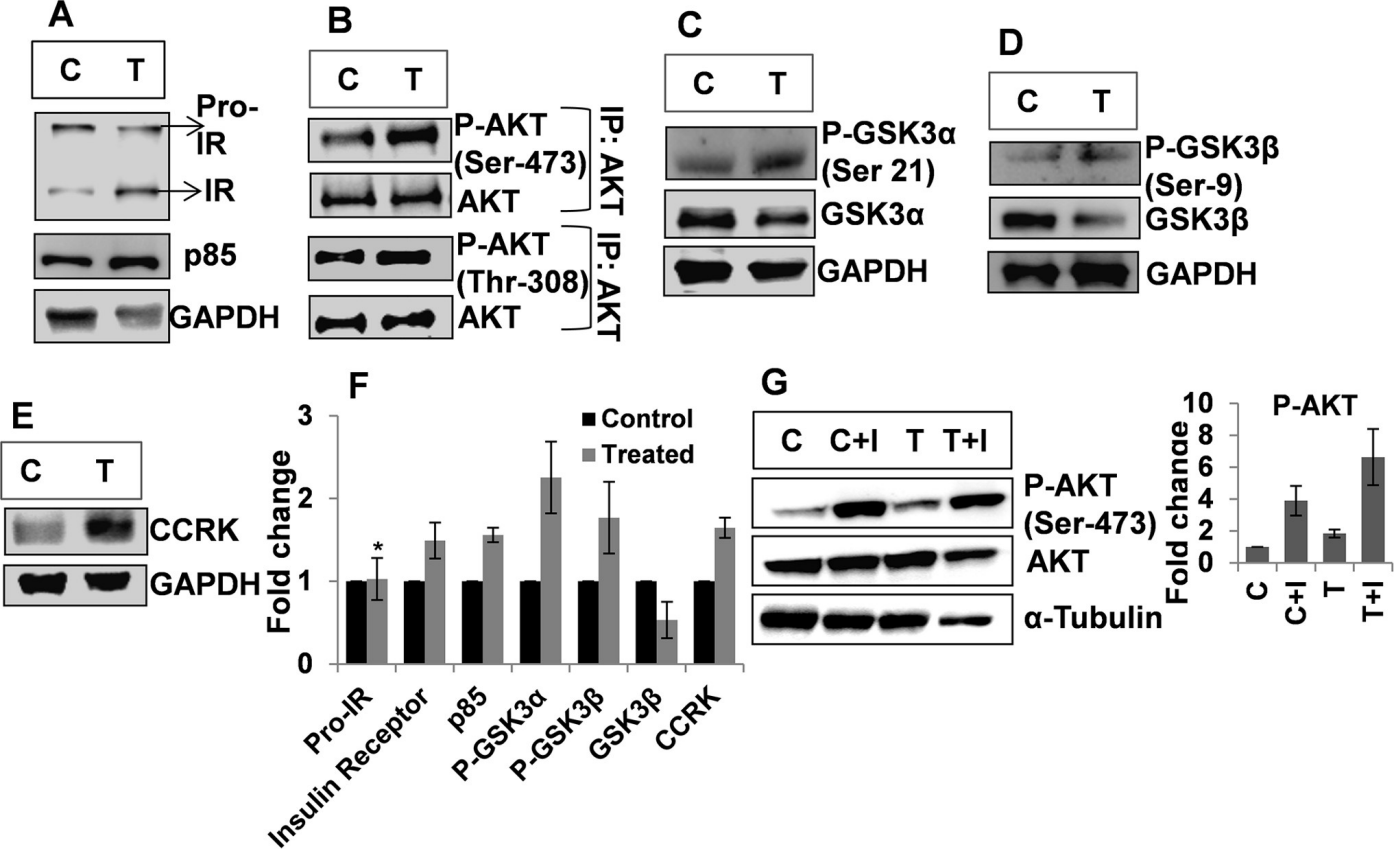
Increased insulin responsiveness in skeletal muscle of treated animals. Fig 2A: Immunoblot for Pro-IR, Insulin Receptor (IR) and p85 in skeletal muscle of control (C) and treated (T) animals. Fig 2B: IP for AKT and Immunoblot for P-AKT (Ser-473 and Thr-308) in skeletal muscle of the control (C) and the treated (T) animals. Fig 2C: Immunoblot for P-GSK3α (Ser-21) in skeletal muscle of control (C) and treated (T) animals. Fig 2D: Immunoblot for P-GSK3β (Ser-9) in skeletal muscle of control (C) and treated (T) animals. Fig 2E: Immunoblot for CCRK in skeletal muscle of control (C) and Treated (T) animals. Fig 2F: Densitometry for Pro-IR, Insulin Receptor (IR), p85, P-GSK3α (Ser-21), P-GSK3β (Ser-9) and P-GSK3β (Ser-9) in skeletal muscle of control (C) and treated (T) animals. The data were analyzed by t-test, data represents mean ±S.D. of 3 independent experiments (n = 3), p< 0.05, * = p>0.05. G: Immunoblot and densitometry for P-AKT (Ser-473) in control and treated animals upon insulin administration, C = control, C+I = control animal with insulin treatment, T = treated, T+I = treated animals with insulin treatment. The data were analysed by two-way repeated measures ANOVA test followed by Bonferroni post hoc analysis; data represents mean ±S.D. of 3 independent experiments (n = 3), p<0.05.

The insulin signalingis initiated by binding of insulin to the Insulin Receptor (IR) on the membrane of insulin responsive cells. We immunoblotted the skeletal muscle tissue lysate of control and treated animals for IR. The treated animals showed significant increase in the IR level as compared to the control ones without any significant change in the Pro-IR levels in the two groups (Fig 2A). O’Neil et al., 2016 have demonstrated that IR plays a critical role in maintaining skeletal muscle mass and IR content increased in mature muscle as compared to the early myoblast stage [17]. The increase in the IR content in the skeletal muscle of the treated animals confirms increased myotube maturation in these animals.

We also found increased p85 levels in skeletal muscle of the treated animals (Fig 2A). To check further for insulin responsiveness in the skeletal muscle of the treated and the control animals, AKT was immuno precipitated from skeletal muscle tissue lysate of the treated and the control animals and immunoblotted for P-AKT (Ser-473 and Thr-308) which are markers for insulin responsiveness [14, 18, 19]. The treated animals showed significant increase in the P-AKT (Ser-473 and Thr-308) levels as compared to the control ones (Fig 2B).

Glycogen Synthase Kinase 3 (GSK3), a serine-threonine kinase, phosphorylates and inactivates Glycogen Synthase, the key enzyme in glycogen synthesis. GSK3 also negatively regulates key signaling molecules like AKT, mTOR and Insulin Receptor Substrate 1 (IRS1). GSK3 perturbs the insulin signaling pathway and its inhibition is decreased in insulin resistance condition [14, 18, 20, 21]. Apart from glycogen synthesis, GSK3β inactivation also plays an important role in myogenic differentiation of atrophied skeletal muscle [22] and resistance training induced skeletal muscle hypertrophy in humans is due to inactivation of GSK3β and activation of mTOR due to Akt phosphorylation and activation [23]. Thus, we checked the inhibitory phosphorylation levels of GSK3α (Ser-21) and GSK3β (Ser-9) in the skeletal muscle of the treated and the control animals. We observed increased P-GSK3α (Ser-21) and P-GSK3β (Ser-9) levels in skeletal muscle of treated animals (Fig 2C and 2D). Collectively, the treated group of animals had increased levels of IR, p85, P-AKT (Ser-473 and Thr-308), P-GSK3α (Ser-21) and P-GSK3β (Ser-9) and decreased levels of total GSK3β as compared to the control ones but there was no significant change in total AKT and GSK3α levels.

In 2011, Feng et al. [24] reported the interaction of an androgen regulated kinase, Cell Cycle Related Kinase or CCRK, with GSK3β. The interaction of CCRK with GSK3β increased Ser-9 phosphorylation. A significant increase in CCRK levels in the skeletal muscles of treated animals as compared to the controls was observed (Fig 2E). Hence a major role is apparently played by the androgen regulated CCRK in GSK3β phosphorylation and inactivation in the skeletal muscle of the treated animals.

Tissue insulin responsiveness is reflected by the P-AKT (Ser-473) levels upon insulin action. To test for insulin responsiveness in the skeletal muscle of the treated and the control animals, we administered exogenous insulin and subsequently checked for P-AKT (Ser-473) levels in their skeletal muscle. The skeletal muscles were isolated 60min after extrinsic insulin administration because in the ITT, we found that the BGL was lowest at the 60min time point. P-AKT (Ser-473) levels were noted to be highest in the treated animals which were also given insulin followed by control animals treated with insulin (Fig 2G). This indicates a scenario of enhanced insulin responsiveness in the skeletal muscle of the treated animals.

The chronic testosterone supplementation could cause global changes and alter glucose homeostasis in the treated animals as testosterone increases myogenesis and the improved glycemic control in the treated animals could be a result of an increased muscle mass. [13]

### Studies on C2C12 cells show that testosterone potentiates insulin signaling in skeletal muscle

Previously, numerous studies have been carried out in which the effect of androgen receptor inhibition, by the use of antagonists or by down regulation, have been studied in numerous cell types and in skeletal muscle. These studies hint to a correlation between the androgen signaling and the insulin signaling pathways. Ma et al., 2017, reported that cyclic mechanical stretch positively regulated proliferation of C2C12 cells, which was directly regulated by PI3K-AKT and MAPK pathways and AR inhibition by flutamide blocked the mechanical stretch regulated proliferation of these cells [25]. In another study using BLA3A cells, it was reported that androgen had a protective effect on H_2_O_2_ treated cells that experienced oxidative stress. This protective effect was mediated through the PI3K-AKT pathway and was reversed in the presence of flutamide [26]. Rossetti et al., 2017, summarized that castration of male mice reduced skeletal muscle P-AKT (Ser-473) and P-mTOR (Ser-2448) level, along with skeletal muscle protein synthesis and this effect was reversed upon exogenous administration of androgen to the to the castrated mice [27].

**Fig 3:**
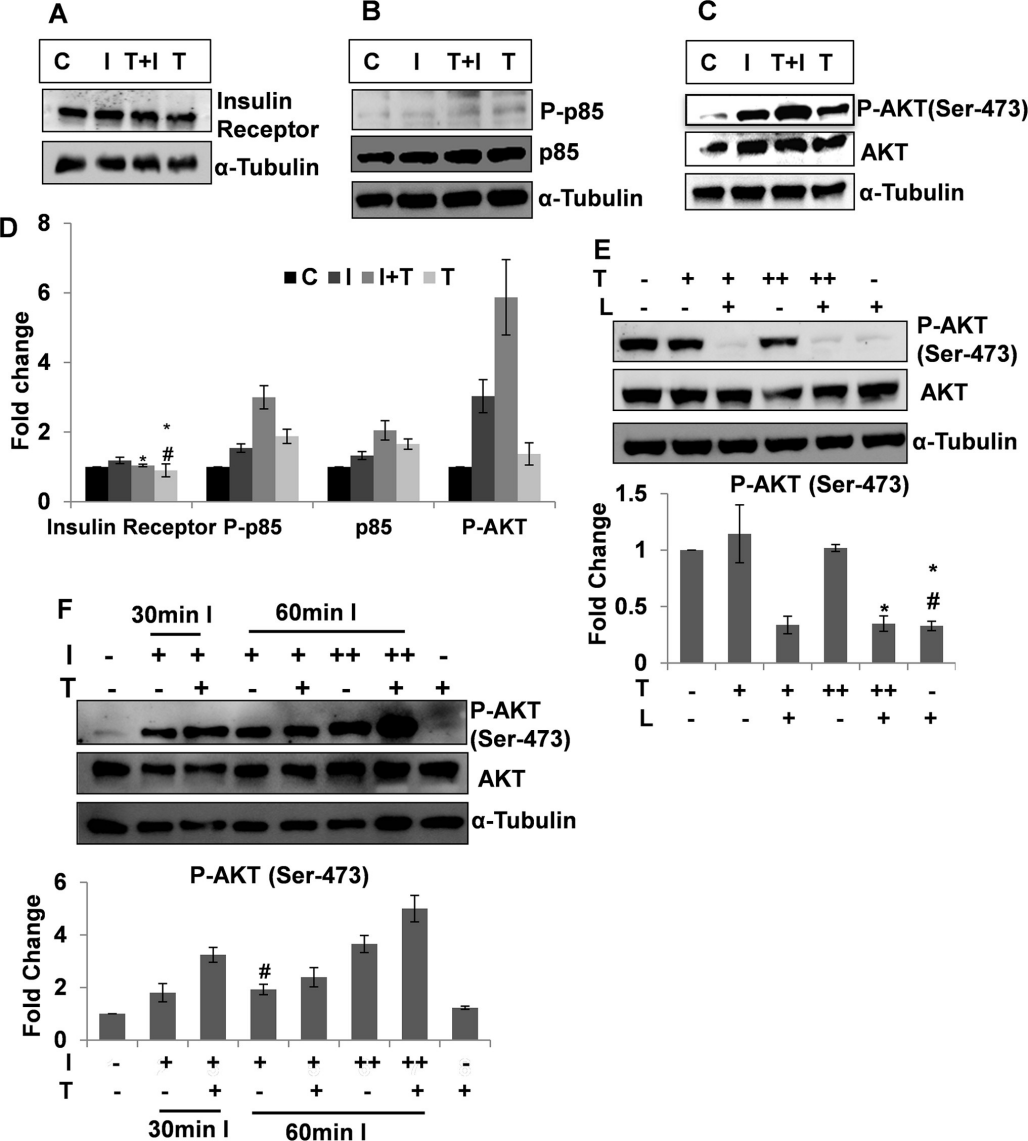
Testosterone potentiates insulin signaling in C2C12 cells. Immunoblot for Insulin Receptor (Fig 3A), P-p85 (Fig 3B) and P-AKT (Ser-473) (Fig 3C) in C2C12 cell lysate, C = control, I = Insulin (10ng/ml), I+T = Insulin (10ng/ml) +Testosterone (50ng/ml), T = Testosterone (50ng/ml). Testosterone pre- treatment was given for 60 min and insulin treatment for 60 min without removing testosterone. Fig 3D: Densitometry for Insulin Receptor, P-p85, p85, P-AKT (Ser-473) in C2C12 cell lysate, C = control, I = Insulin (10ng/ml), I+T = Insulin (10ng/ml) +Testosterone (50ng/ml), T = Testosterone (50ng/ml). Testosterone pre- treatment was given for 60 min and insulin treatment for 60 min without removing testosterone. The data were analyzed by two-way repeated measures ANOVA test followed by Bonferroni post hoc analysis; data represents mean ±S.D. of 3 independent experiments (n = 3), p<0.05, * = no significant change as compared to C; # = no significant change as compared to I+T. Fig 3E: Immunoblot and densitometry for P-AKT (Ser-473) in C2C12 cells upon testosterone and/ or PI3K inhibitor treatment, T (+) = 50ng/ml Testosterone, T (++) = 100ng/ml Testosterone, L (+) = 10μM LY294002; LY294002 added 15min prior to testosterone treatment, testosterone treatment was given for 60 min without removing LY294002; The data were analyzed by two-way repeated measures ANOVA test followed by Bonferroni post hoc analysis; data represents mean ±S.D. of 3 independent experiments (n-3), p<0.05, * = no significant change as compared to T(+)L(+); # = no significant change as compared to T(++)L(+). Fig 3F: Immunoblot and densitometry for P-AKT (Ser-473) in C2C12 cells, I (+) = Insulin (10ng/ml), I(++) = Insulin (250ng/ml), T(+) = Testosterone (50ng/ml). Testosterone treatment to cells for 120 min and 30min I = insulin treatment for 30min, 60min I = insulin treatment for 60 min, without removing testosterone; The data were analyzed by two-way repeated measures ANOVA test followed by Bonferroni post hoc analysis; data represents mean ±S.D. of 3 independent experiments (n-3), p<0.05; # = no significant change as compared to I(+)T(-), 30min I.

To study the effect of testosterone specifically on the insulin signalling PI3K-AKT pathway in skeletal muscles we conducted *in vitro* experiments in C2C12 myocyte cell line. C2C12 cells were subjected to an acute exposure of testosterone to minimise the effect of mitogenic and differentiation activities of testosterone on the C2C12 cells. C2C12 cells were incubated with or without insulin or testosterone or both and the cell lysates were then probed for IR, p-85 and AKT. No significant change in the IR levels of the unstimulated, both insulin and testosterone treated and only testosterone treated cells were found though the only insulin treated cells showed some increase in IR (Fig 3A). This finding is in contrast to the *ex vivo* finding, where the skeletal muscle of testosterone treated animals showed increased IR levels, indicating increased myogenesis. The 120 min testosterone treatment was not sufficient for the testosterone to demonstrate myogenic actions and hence, the IR levels showed no significant change. There was a significant increase in P-p85 levels in cells treated with insulin compared to the unstimulated controls. The highest level of P-p85 was observed in cells treated with both testosterone and insulin followed by the cells treated with only testosterone (Fig 3B). Interestingly a significant increase in p85 level was observed in cells treated with both insulin and testosterone and only testosterone as compared to the only insulin treated and unstimulated cells. An increase in p85 gene expression in the skeletal muscle of the treated animals was observed in the microarray analysis of gene expression (S2 Table), consistent with its regulation by an androgen [28].

Correspondingly, cells treated with both testosterone and insulin, showed highest level of P-AKT (Ser-473), followed by that of only insulin stimulated ones or testosterone treated cells (Fig 3C). This indicates a combined action of insulin and testosterone on the skeletal muscle insulin responsiveness and substantiates the findings of *in vivo* studies.

Next to check whether testosterone mediated activation of AKT is PI3K dependent or not, we pre-treated cells with PI3K inhibitor LY294002, and then added testosterone. PI3K inhibition caused marked decrease in P-AKT (Ser-473) levels, irrespective of the presence or absence of testosterone (Fig 3E). The increase in testosterone concentration could not reverse this effect. This confirms that the testosterone mediated enhancement in skeletal muscle insulin sensitivity of the treated animals is dependent on PI3K/AKT pathway.

It was also observed that cells treated with both testosterone and 250 ng/ml insulin for 60 min had the highest level of P-AKT (Ser-473) followed by the cells treated with only 250ng/ml insulin for 60 min (Fig 3E). The P-AKT (Ser-473) levels of cells treated with testosterone and 10ng/ml insulin for 30 min or testosterone and 10ng/ml insulin for 60 min were significantly less than that of the cells treated with both testosterone and 250ng/ml insulin for 60 min (Fig 3F). This reinforces above findings about the potentiating effect on insulin signaling the skeletal muscle.

### Testosterone inactivates GSK3α in C2C12 cells in a PI3K/AKT pathway dependent manner but does not act in synergy with insulin to inactivate GSK3β

**Fig 4:**
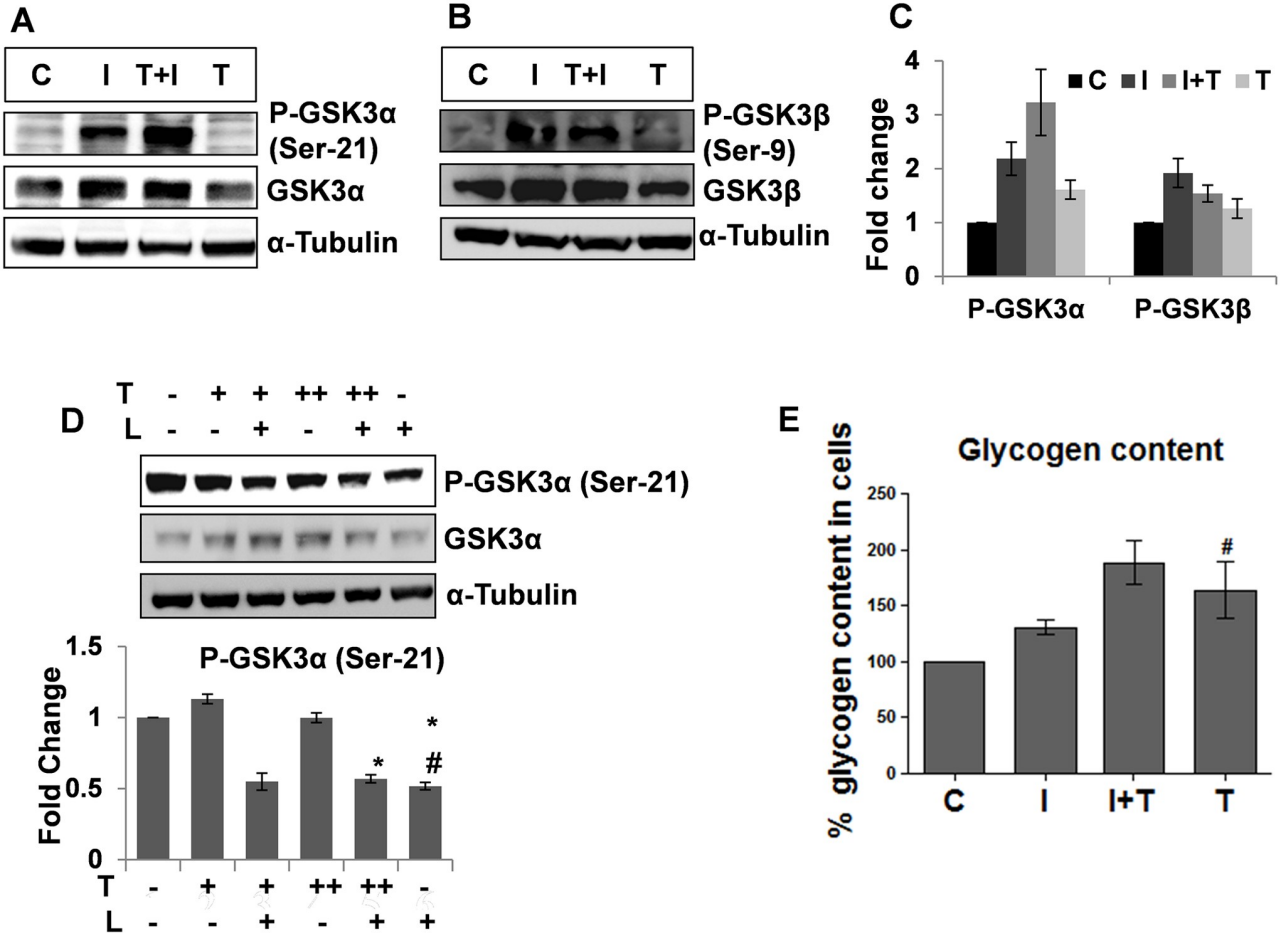
Testosterone inactivates GSK3α in a PI3K/AKT pathway dependent manner but not GSK3β in C2C12 cells. Immunoblot for P- GSK3α (Ser-21) (Fig 4A) and P- GSK3β (Ser-9) (Fig 4B) in C2C12 cells, C = control, I = Insulin (10ng/ml), I+T = insulin (10ng/ml) +testosterone (50ng/ml), testosterone (50ng/ml). Testosterone pre- treatment was given for 60 min and insulin treatment for 60 min, without removing testosterone. Fig 4C: Densitometry for P- GSK3α (Ser-21) and P- GSK3β (Ser-9) in C2C12 cells, C = control, I = Insulin (10ng/ml), I+T = insulin (10ng/ml) +testosterone (50ng/ml), testosterone (50ng/ml). Testosterone pre- treatment was given for 60 min and insulin treatment for 60 min, without removing testosterone. The data were analyzed by two-way repeated measures ANOVA test followed by Bonferroni post hoc analysis; data represents mean ±S.D. of 3 independent experiments (n-3), p<0.05. Fig 4D: Immunoblot and densitometry for P- GSK3α (Ser-21) in C2C12 cells upon testosterone and/ or PI3K inhibitor treatment, T (+) = 50ng/ml testosterone, T (++) = 100ng/ml testosterone, L (+) = 10μM LY294002; LY294002 added 15min prior to testosterone treatment, testosterone treatment was given for 60 min without removing LY294002; The data were analyzed by two-way repeated measures ANOVA test followed by Bonferroni post hoc analysis; data represents mean ±S.D. of 3 independent experiments (n-3), p<0.05, * = no significant change as compared to T(+)L(+)# = no significant change as compared to T(++)L(+). Fig 3E: Glycogen content assay in C2C12 cells. C = control, I = insulin (10ng/ml), I+T = insulin (10ng/ml) +testosterone (50ng/ml), T = testosterone (50ng/ml). Testosterone pre-treatment was given for 60 min and insulin treatment for 60 min, without removing testosterone. The data were analyzed by two-way repeated measures ANOVA test followed by Bonferroni post hoc analysis; data represents mean ±S.D. of 5 independent experiments (n = 5), p<0.05, # = no significant change as compared to I+T.

Further to study the effect of testosterone on GSK3α and GSK3β inactivation in the skeletal muscle, we treated C2C12 cells with insulin or testosterone or both or nothing. C2C12 cells treated with both testosterone and insulin had the highest P-GSK3α (Ser-21) levels as compared to the unstimulated or only insulin or testosterone stimulated ones (Fig 4A). The immunoblot analysis for P-GSK3β (Ser-9) in C2C12 cells showed significant increase in P-GSK3β (Ser-9) levels in cells treated with testosterone and both insulin and testosterone as compared to the unstimulated ones (Fig 4B), while, the levels of P-GSK3β (Ser-9) in cells treated with only insulin were the highest. We also studied the role of the PI3K/AKT pathway in the testosterone mediated GSK3α inactivation. It was observed that upon PI3K inhibition, testosterone alone is unable to inactivate GSK3α (Fig 4D). Thus, the testosterone mediated GSK3α inactivation in skeletal muscle is dependent on the activation of PI3K/AKT pathway. Further determination of glycogen levels in C2C12 cells incubated with or without insulin or testosterone or both showed that glycogen content was maximum in the cells treated with both insulin and testosterone (Fig 4E). Testosterone treated cells showed no significant difference in the glycogen content as compared to the both insulin and testosterone treated ones. This could possibly be due to the reduced levels of GSK3β in cells treated with testosterone.

**Fig 5:**
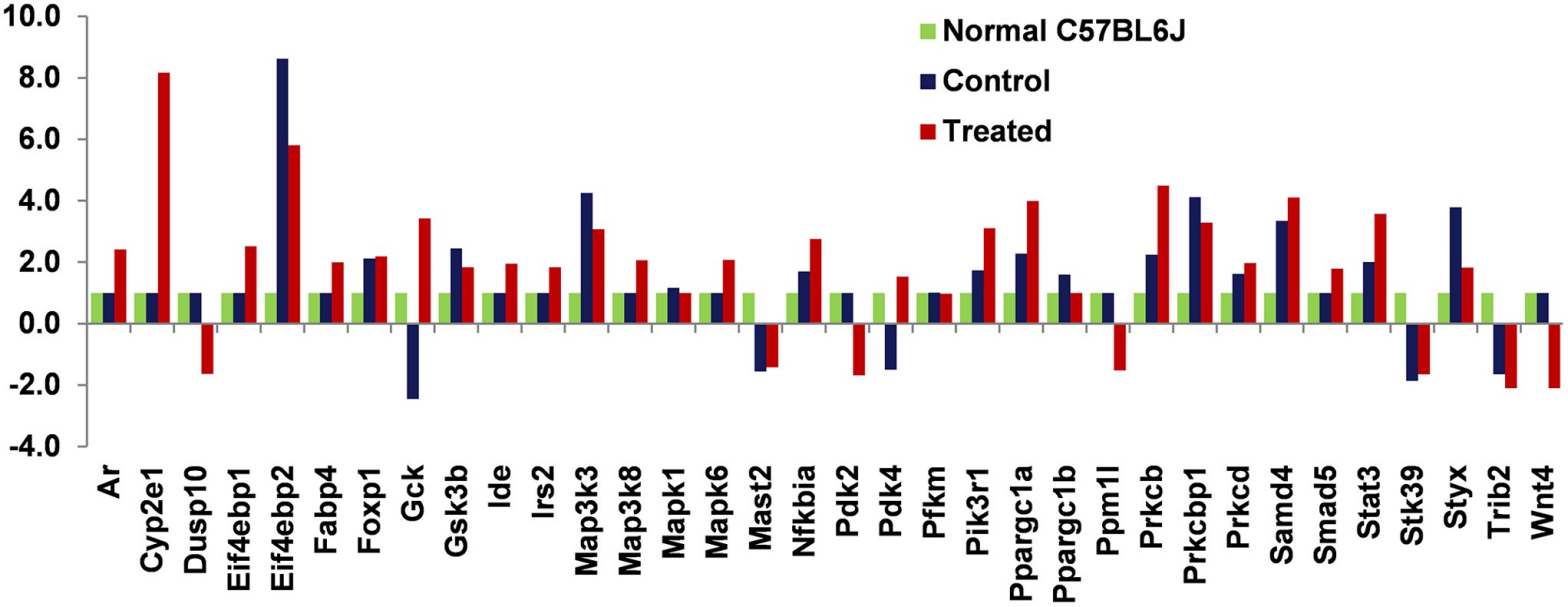
Microarray analysis of differential expression of genes in skeletal muscle of treated and control animals, with respect to age matched, normal chow fed male C57BL6J mice. Microarray analysis showing differences in the expression levels of various genes involved in glycemic control in the skeletal muscle of the treated and control animals after 32 weeks of treatment, with respect to age matched, normal chow fed male C57BL6J mice.

However, on the other hand the GSK3β expression is regulated by testosterone as depicted by immunoblot analysis for GSK3β and microarray analysis (Fig 5) of the skeletal muscle, insulin inactivates GSK3β in a potent manner.

### Differential expression analysis of genes involved in insulin responsiveness or related pathways in skeletal muscle of the treated and the control animals in comparison to normal chow fed age matched male C57BL6J mice

Heinlein and Chang, 2002, summarized the genomic and non-genomic mode of action of AR. The genomic mode of action directly affects the expression of androgen responsive genes while the non-genomic AR action can activate Protein Kinase A, Protein Kinase C and MAPK, without affecting the gene expression [29]. In our previous study [9], we reported that AR-FOXO1 interaction led to the degradation of FOXO1 to a non-functional product that resulted in reduced hepatic glucose output in the treated mice.

Microarray analysis was done for the differential expression of genes involved in insulin responsiveness and other related pathways in the skeletal muscle of treated and control animals in comparison to normal chow fed age matched male C57BL6J mice (S2 and S3 Tables). The histogram in Fig 5 represents the genes which have been deduced to play a role in the testosterone mediated alteration of glucose homeostasis in the skeletal muscle of the treated animals. Bolton et al., 2007, used RNA expression profiling to identify Androgen Regulated Genes (ARGs) and like us, they reported the positive induction of IRS2 and NFKBIA in response to androgen action [30]. In another study, it was reported that skeletal muscle AR deficiency in males, resulted in reduced PGC-1α levels [5]. The transcriptome analysis in male mice with AR deficient β-islet, by Xu et al., 2017, revealed that AR action influenced JAK-STAT, Insulin signaling and MAPK pathways in these cells [31].

From our experiments involving immunoblot analysis of isolated tissue and cultured cell lysate, we found that testosterone acted in insulin dependent manner to activate AKT and inactivate GSK3α in the skeletal muscle. The microarray data analysis showed no significant change in AKT or GSK3α gene expression in the skeletal muscle upon testosterone supplementation. The changes in the phosphorylation level and not in the expression level for AKT or GSK3α lead to an enhanced glycemic control in the skeletal muscle in the treated animals. On the contrary, we found an increase in p85 subunit of PI3K and a decrease in GSK3β gene expression upon testosterone administration, which was also validated in the immunoblot analysis, in the skeletal muscle of the treated animals. The microarray analysis also revealed an increase in expression of IRS2 in the testosterone treated skeletal muscles. Thus, testosterone regulates the expression of p85, GSK3β and IRS2 in the skeletal muscle to improve insulin responsiveness.

## In summary

Testosterone supplementation improved glycemic control in male HFD fed T2DM animals. The increased myogenesis in the treated animals due to the anabolic steroid, testosterone, indicated by the increased IR levels, resulted in enhanced glucose uptake and utilization by them, accounting for the improved overall glycemic control in the treated animals. A detailed investigation showed that testosterone potentiates insulin signaling the skeletal muscle by positively regulating p85 gene expression. In our previous study [9], we showed that testosterone supplementation reduced hepatic glucose output despite increasing hepatic insulin resistance. The IR level in the liver of treated and control animals showed no significant change (p>0.05) (S15 Fig) despite the development of hepatic insulin resistance and this is in contrast to the increased IR level in the skeletal muscle of the treated animals as compared to the control. The diminished hepatic gluconeogenesis is demonstrated to emanate from a reduced level of PEPCK. We also compared the insulin responsiveness of the treated and the control animals, after 32 weeks of treatment, with age matched normal chow fed male C57BL6J mice. We found that the treated animals were more responsive to the administered insulin as compared to the age matched normal chow fed male C57BL6J mice (S16 Fig). The reduced hepatic glucose output due to testosterone supplementation to the HFD fed males caused hypoglycaemia in the treated animals in response to the extrinsic insulin.

Thus, the two key organs involved in glucose homeostasis, skeletal muscle and liver, respond in different ways to the same stimulus, testosterone. The skeletal muscle and the liver showed striking difference in the regulation of GSK3α, which is an important component in the glucose homeostatic pathway in both the tissues as the testosterone mediated GSK3α inhibition in the skeletal muscle is dependent on the PI3K/AKT pathway, whereas in the liver it is PI3K/AKT independent.

## Conclusion

Overall testosterone treatment in HFD fed T2DM male animals improves glycemic control, but the outcomes at tissue level are strikingly different. While testosterone stimulates the skeletal muscle insulin signaling pathway to improve glucose homeostasis, in the liver it acts independent of the insulin signaling pathway to reduce hepatic glucose output, both of which co-operate together to improve the overall glycemic control. Yu et. al., 2014, discussed the effects of Androgen Deprivation Therapy (ADT) in patients with Prostate cancer (PC), which resulted in severe testosterone deficiency in these patients. These patients developed different components of metabolic syndrome including, insulin resistance, diabetes, obesity and cardiovascular complications. They also mention that the molecular mechanisms involving the androgen mediated regulation of energy metabolism in men, possibly involves multiple factors along with tissue cross-talk among insulin responsive tissues [32].

Though our study and other reports demonstrate that testosterone positively regulates glycaemic control in males, one needs to tread cautiously about the use of testosterone in a therapeutic regime to treat T2DM [33].

## Supporting information

S1 Fig Full blot images for Fig 2A. (TIF)

S2 Fig Full blot images for Fig 2B. (TIF)

S3 Fig Full blot images for Fig 2C. (TIF)

S4 Fig Full blot images for Fig 2D. (TIF)

S5 Fig Full blot images for Fig 2E. (TIF)

S6 Fig Full blot images for Fig 2G. (TIF)

S7 Fig Full blot images for Fig 3A. (TIF)

S8 Fig Full blot images for Fig 3B. (TIF)

S9 Fig Full blot images for Fig 3C. (TIF)

S10 Fig Full blot images for Fig 3E. (TIF)

S11 Fig Full blot images for Fig 3F. (TIF)

S12 Fig Full blot images for Fig 4A. (TIF)

S13 Fig Full blot images for Fig 4B. (TIF)

S14 Fig Full blot images for Fig 4D. (TIF)

S15 Fig Immuno blot and densitometry for Insulin Receptor (IR) in liver of control (C) and treated (T) animals. The data were analyzed by t-test, data represents mean ±S.D. of 3 independent experiments (n = 3), * = p>0.05.
(TIF)

S16 Fig ITT of the treated (T2DM Treated), control (T2DM Control) and age matched normal chow fed male C57BL6J (Normal C57BL6J) mice after 32 weeks of treatment. The data were analysed by two-way repeated measures ANOVA test followed by Bonferroni post hoc analysis; data represents mean ±S.D. n = 8, N = 2, p< 0.05, * = no significant difference between the groups.
(TIF)

S1 Table Serum levels of hormones and analytes involved in glycemic control. N = Normal Chow fed 46 weeks old C57BL6J male mice; C = HFD fed age matched C57BL6J male mice; T = HFD fed age matched C57BL6J male mice supplemented with testosterone. n = 10, p<0.005. # = p>0.1 as compared to C.
(DOCX)

S2 Table Microarray analysis showing difference in expression level of different kinases in the skeletal muscle of the treated (T) and control (C) animals after 32 weeks of treatment, with respect to age matched, normal chow fed male C57BL6J mice (N). Blank boxes in table indicate no change in expression level as compared to N; F.C. = Fold Change.
(DOCX)

S3 Table Microarray analysis showing difference in the expression level of different genes in the skeletal muscle of the treated (T) and control (C) animals after 32 weeks of treatment, with respect to age matched, normal chow fed male C57BL6J mice (N). Blank boxes in table indicate no change in expression level as compared to N; F.C. = Fold Change.
(DOCX)
