# Supplementary figures and images for: Testosterone supplementation improves insulin responsiveness in HFD fed male T2DM mice and potentiates insulin signaling in the skeletal muscle and C2C12 myocyte cell line
Source: PLoS One. 2019 Nov 6;14(11):e0224162. doi: 10.1371/journal.pone.0224162 (PMC6834245; doi:10.1371/journal.pone.0224162)

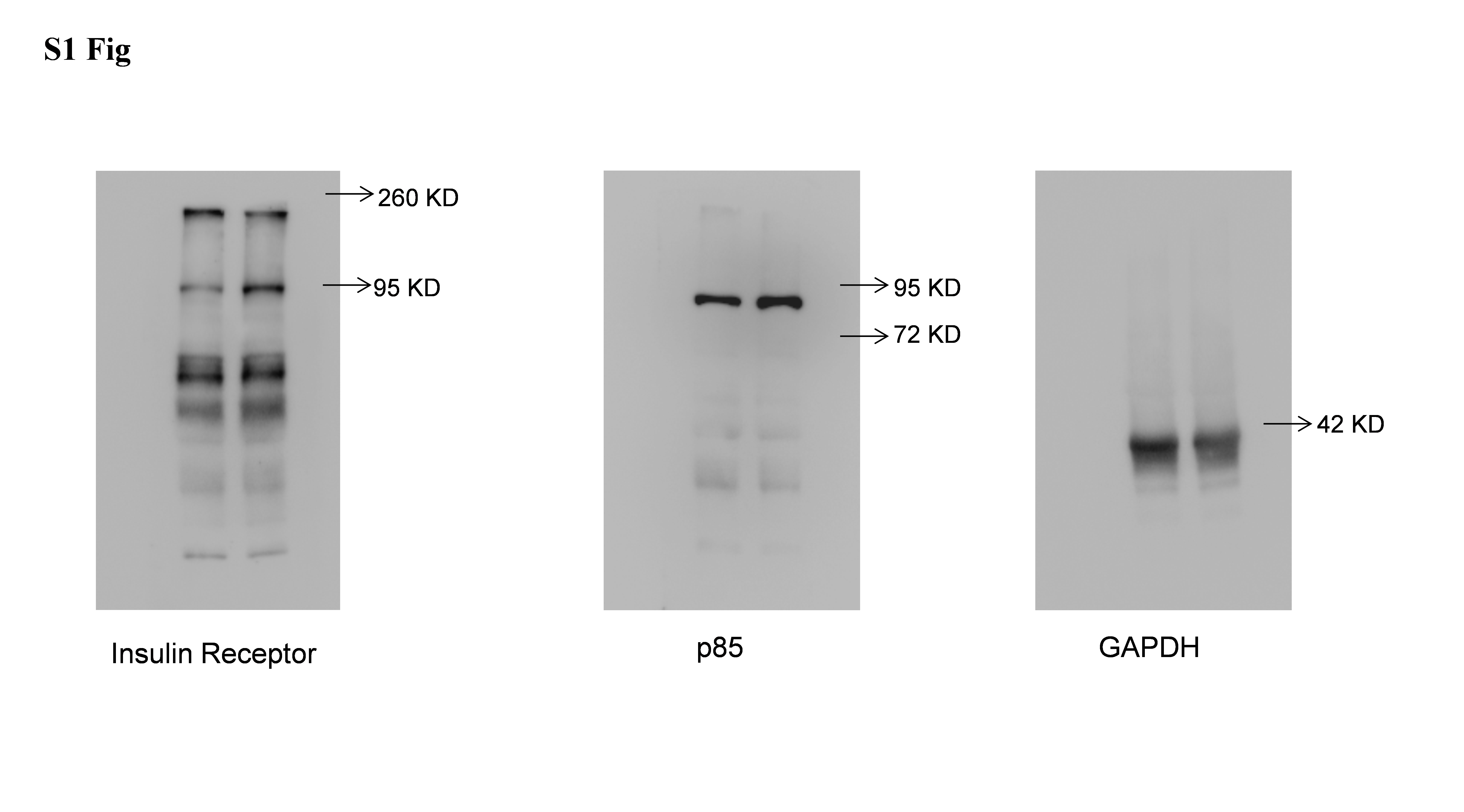

Supplement: S1 Fig — (TIF) [file pone.0224162.s001.tif]

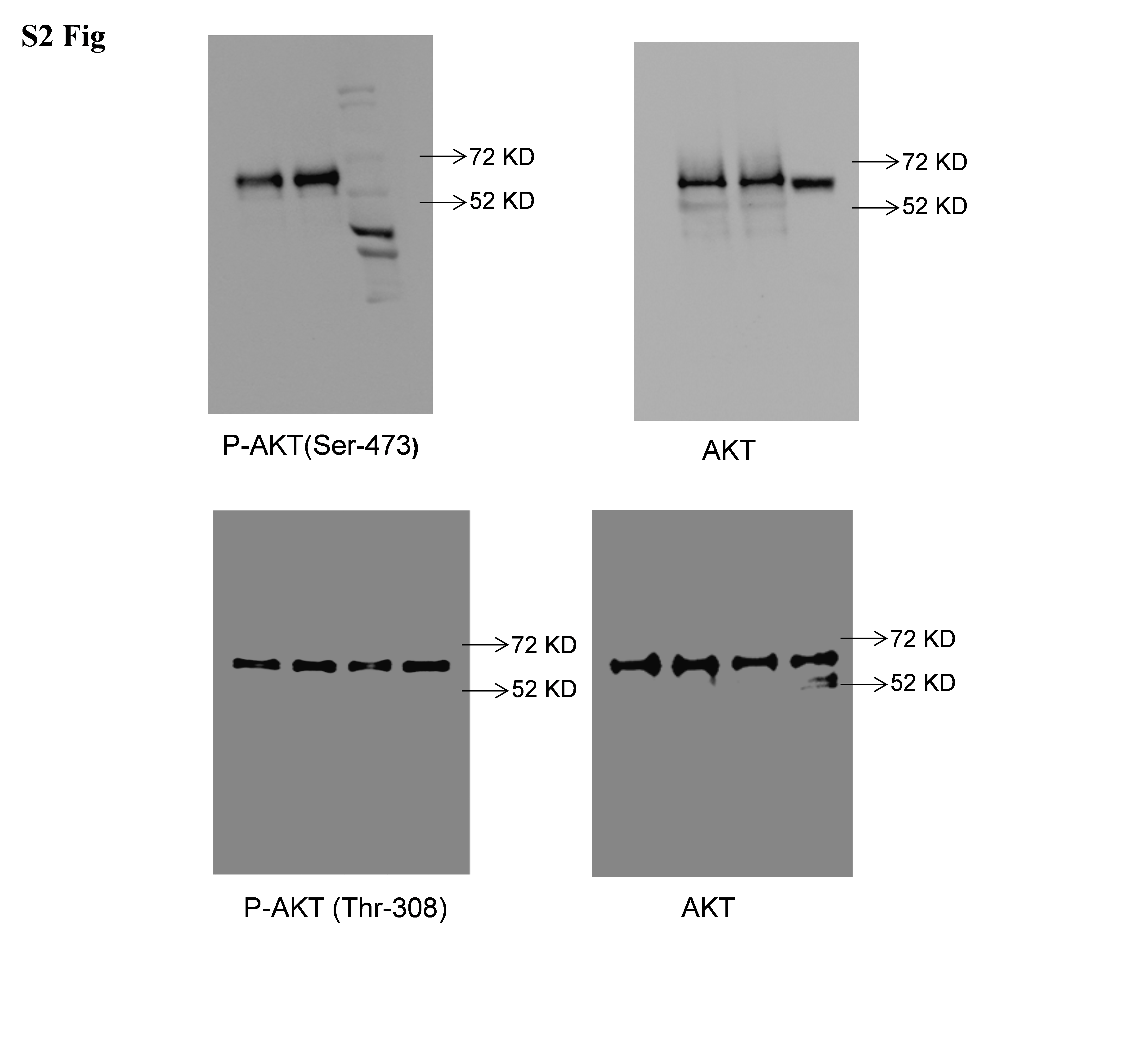

Supplement: S2 Fig — (TIF) [file pone.0224162.s002.tif]

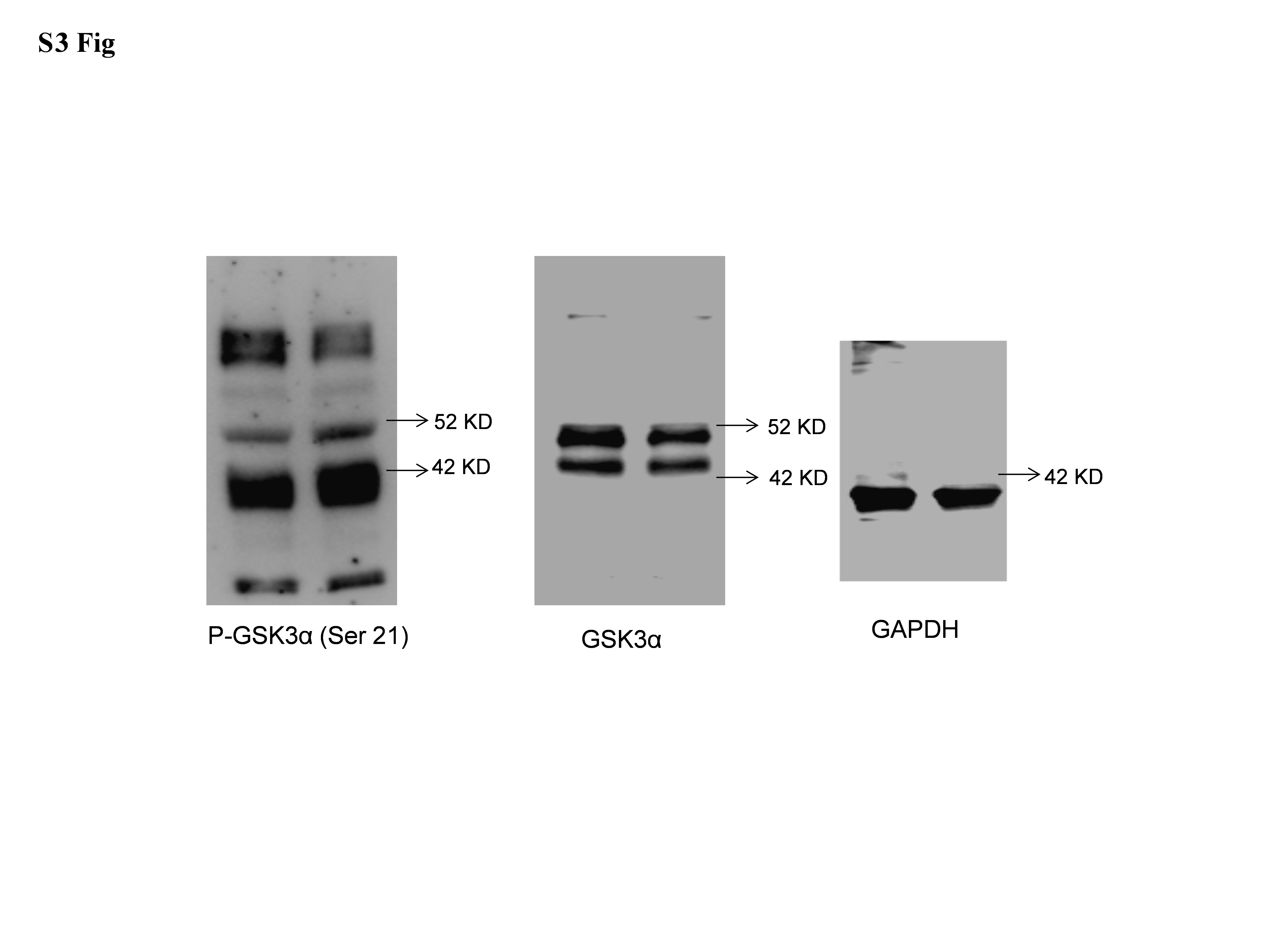

Supplement: S3 Fig — (TIF) [file pone.0224162.s003.tif]

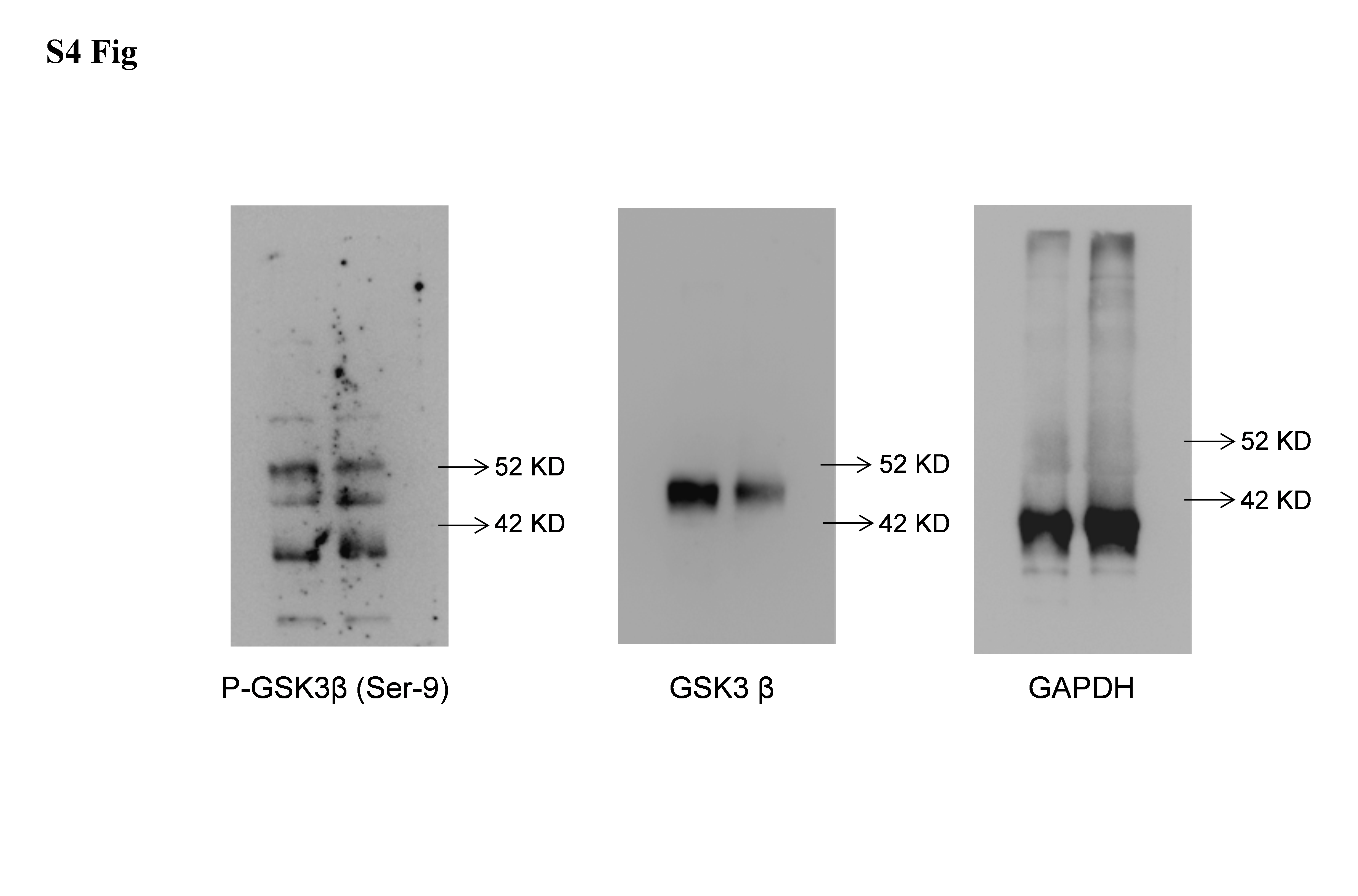

Supplement: S4 Fig — (TIF) [file pone.0224162.s004.tif]

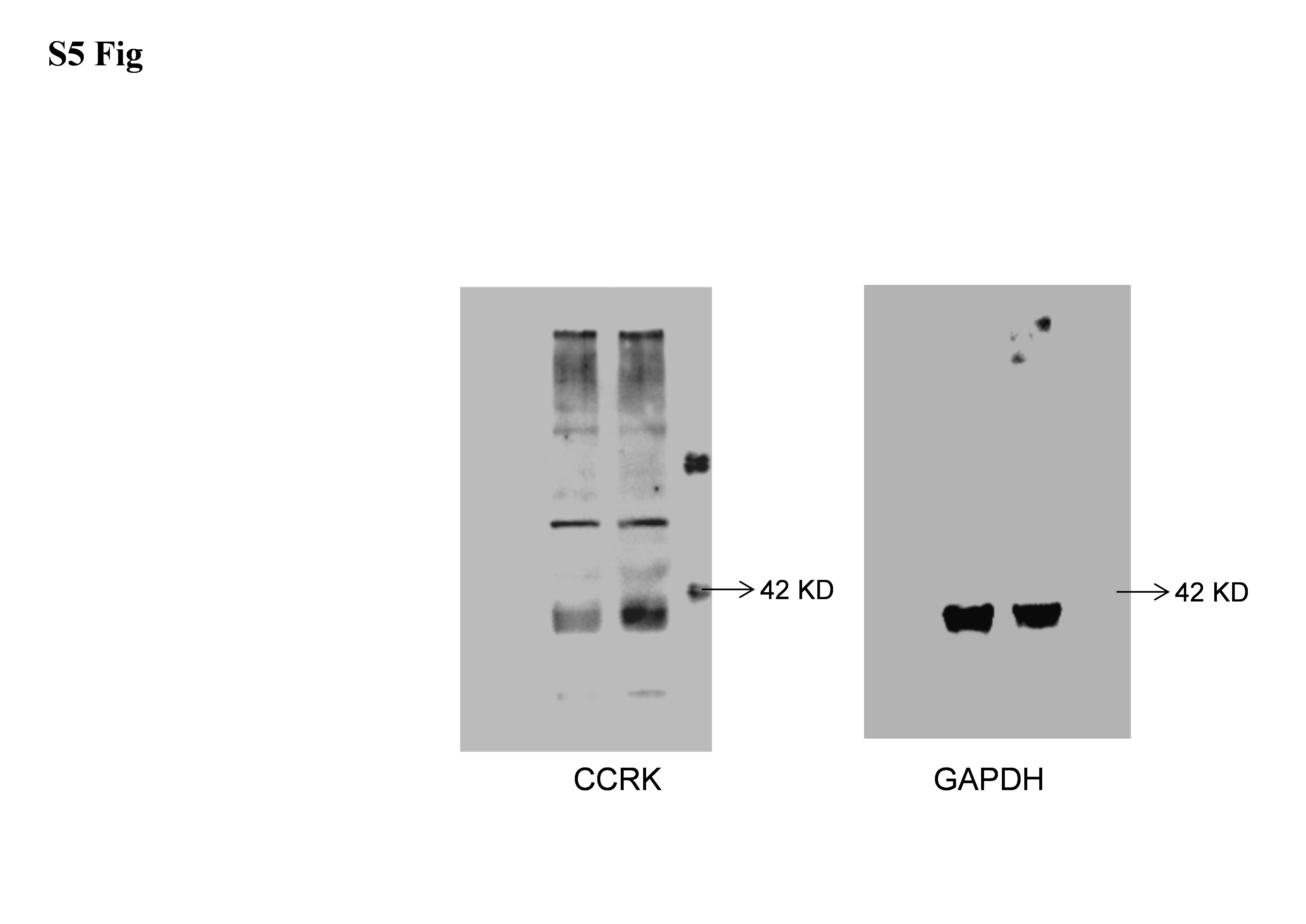

Supplement: S5 Fig — (TIF) [file pone.0224162.s005.tif]

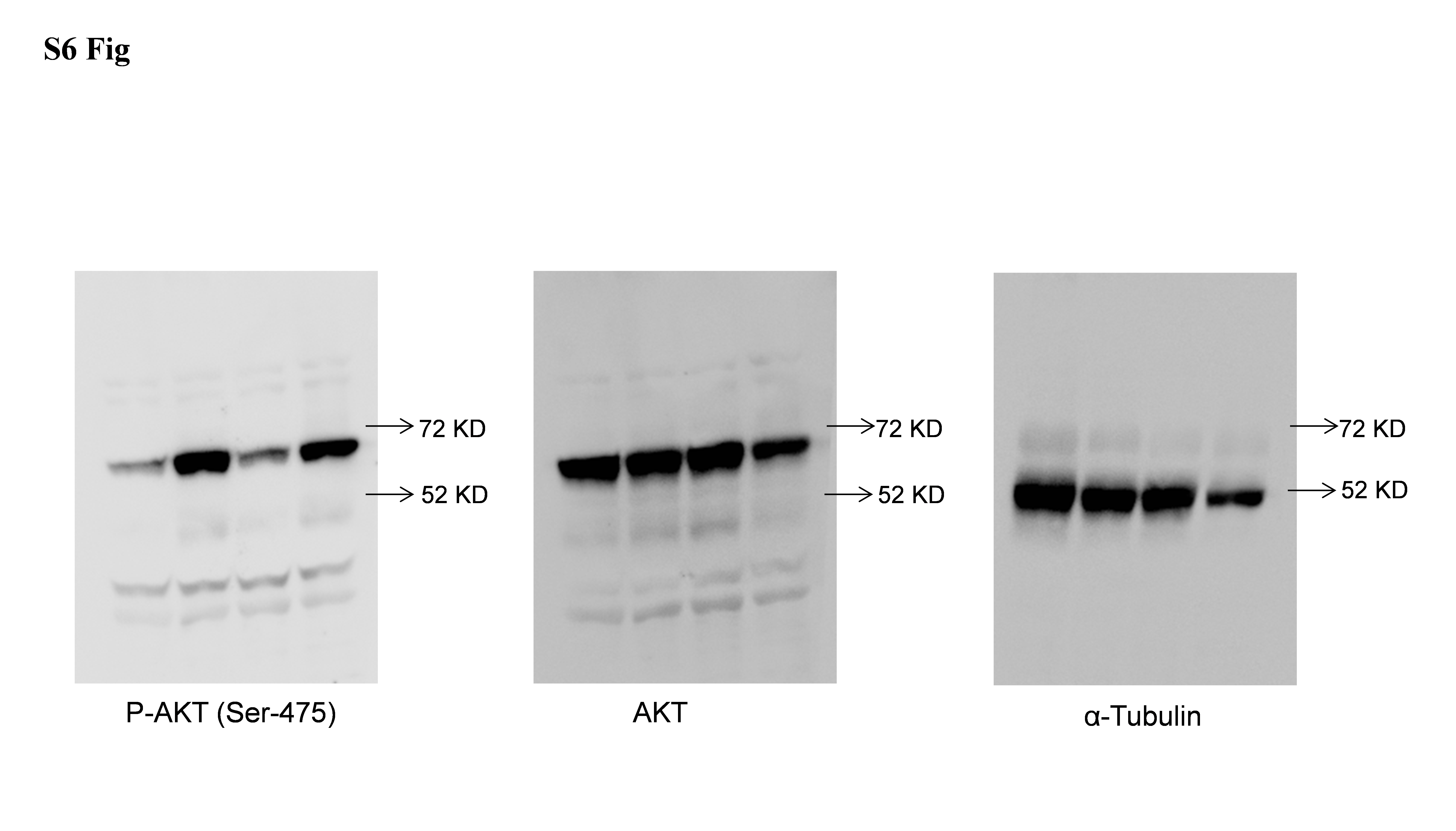

Supplement: S6 Fig — (TIF) [file pone.0224162.s006.tif]

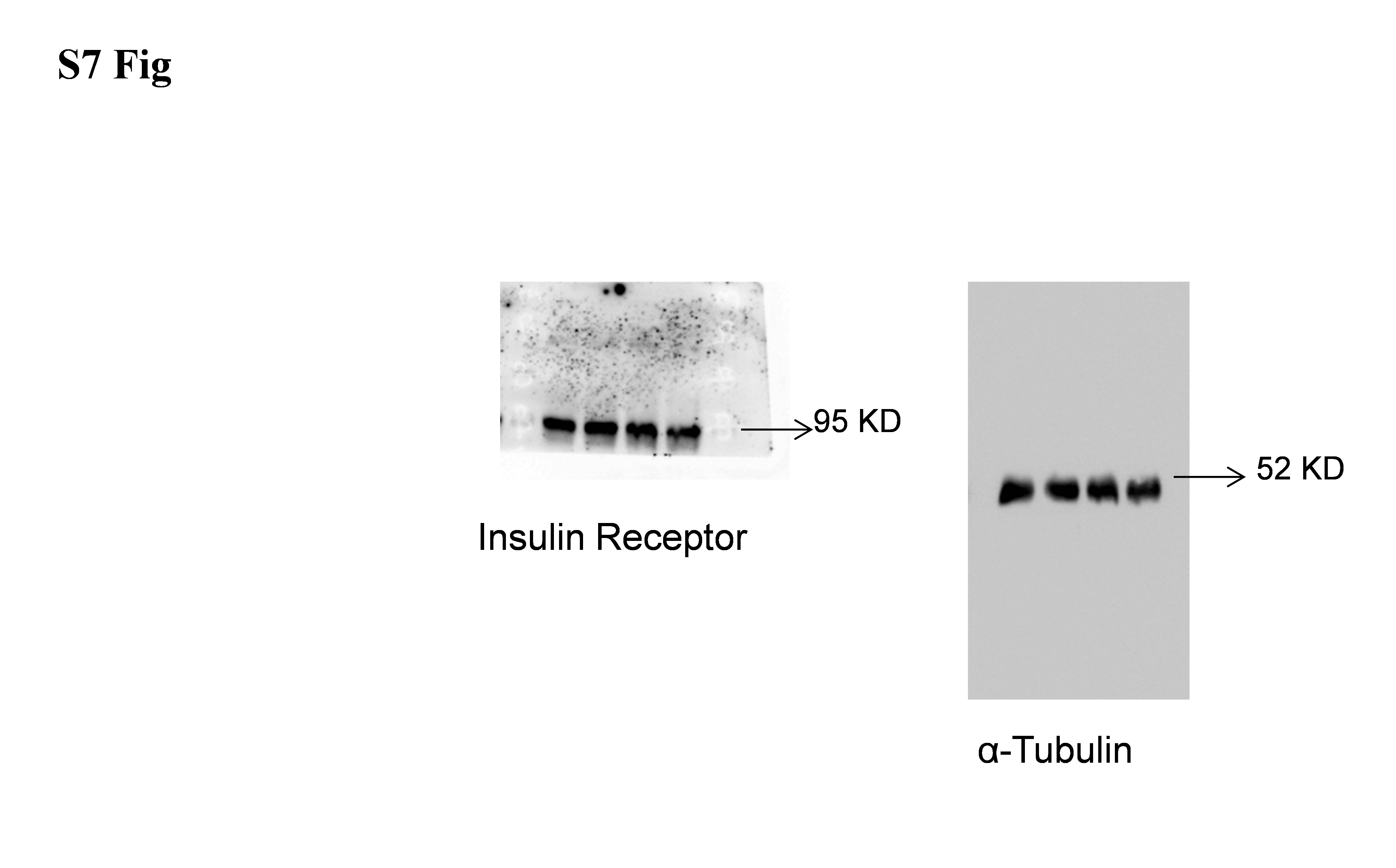

Supplement: S7 Fig — (TIF) [file pone.0224162.s007.tif]

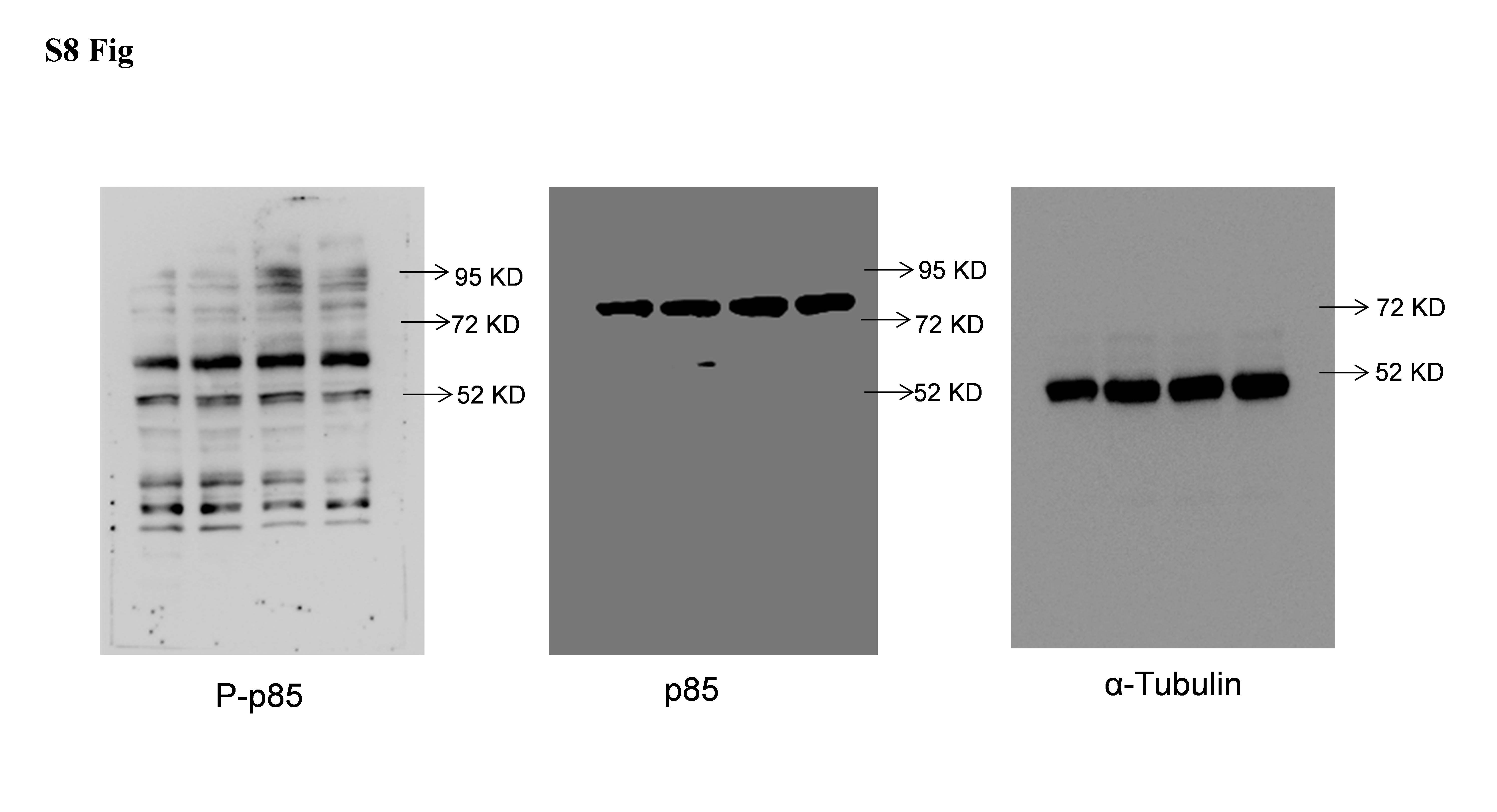

Supplement: S8 Fig — (TIF) [file pone.0224162.s008.tif]

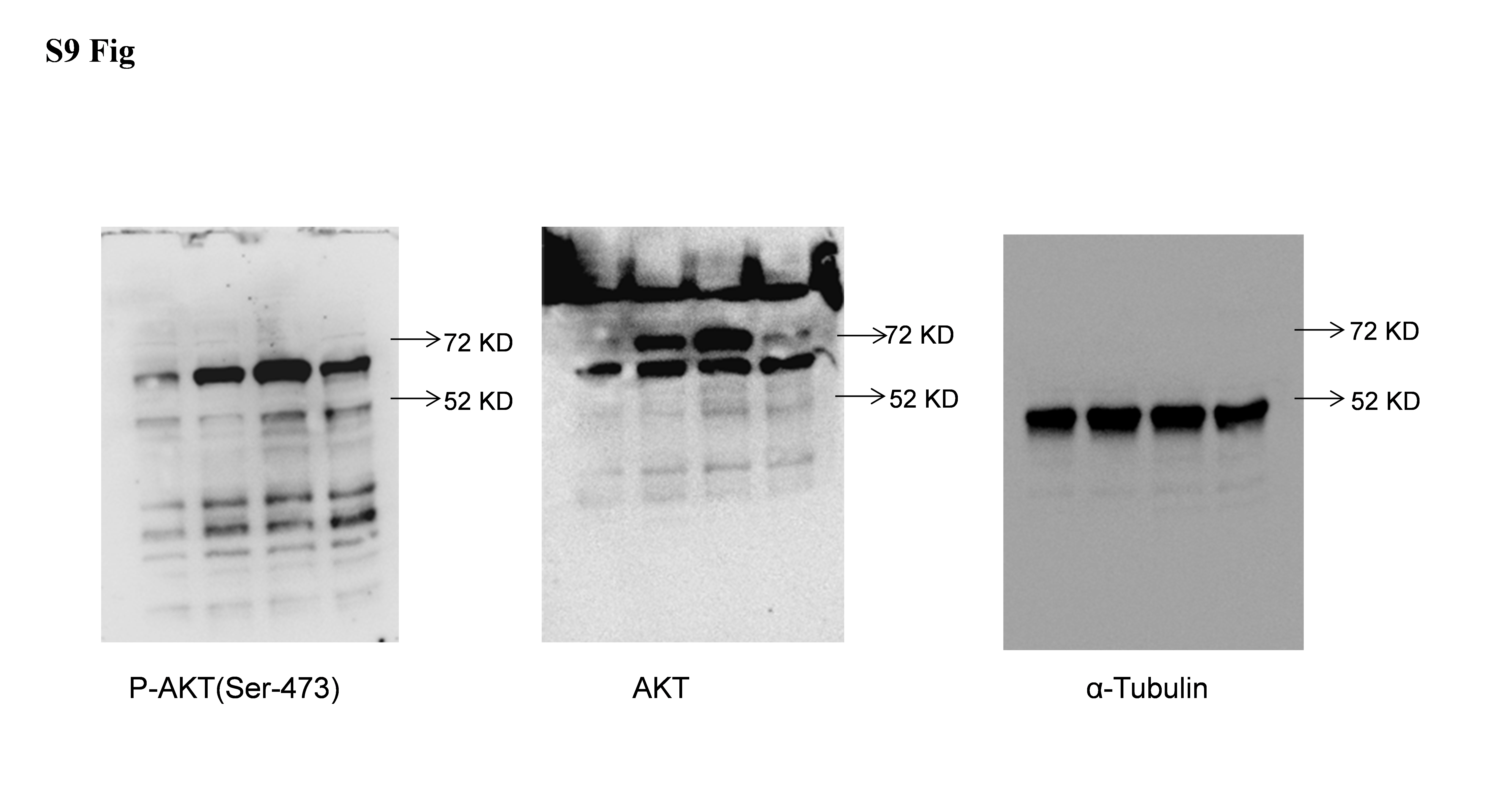

Supplement: S9 Fig — (TIF) [file pone.0224162.s009.tif]

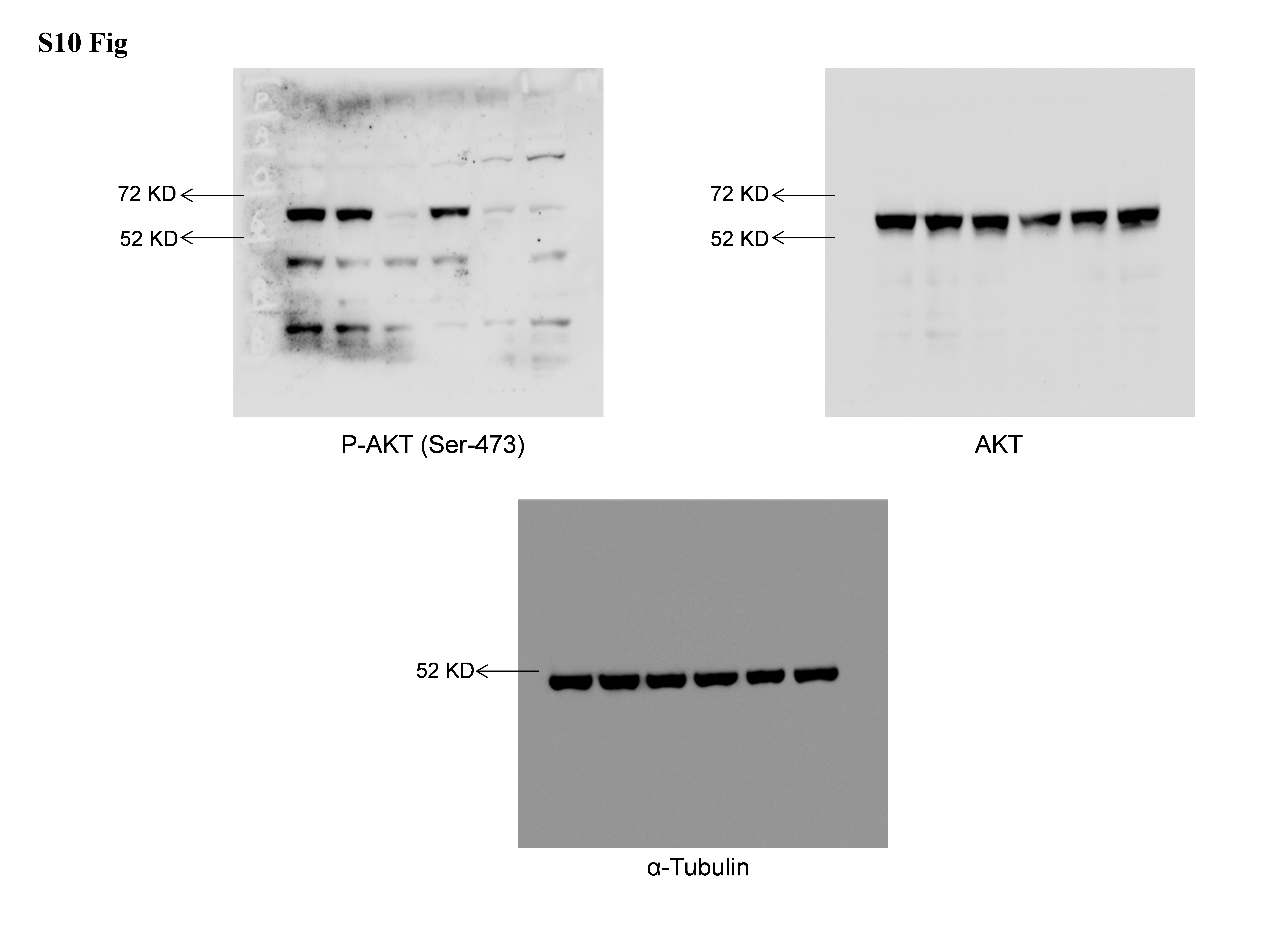

Supplement: S10 Fig — (TIF) [file pone.0224162.s010.tif]

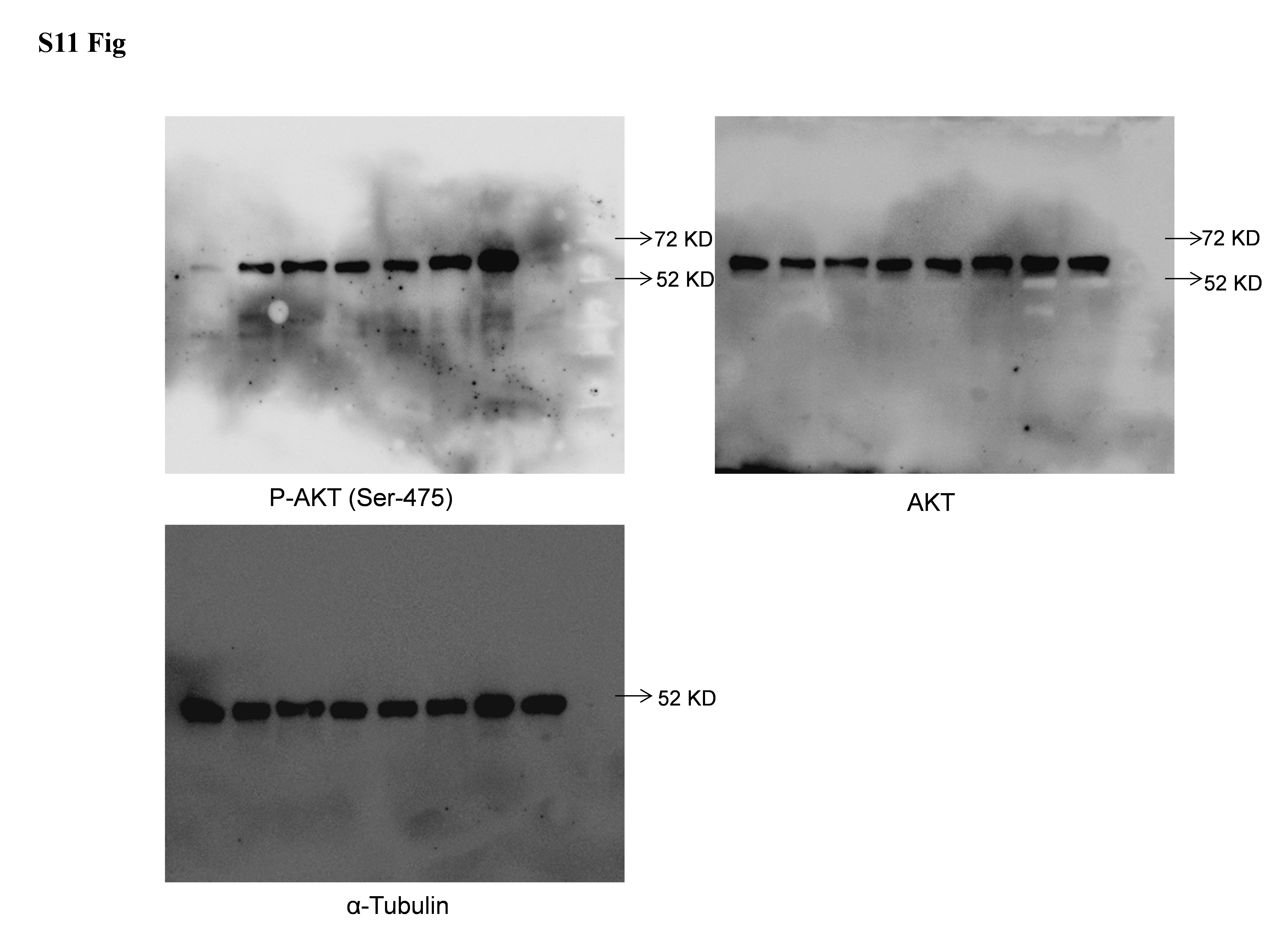

Supplement: S11 Fig — (TIF) [file pone.0224162.s011.tif]

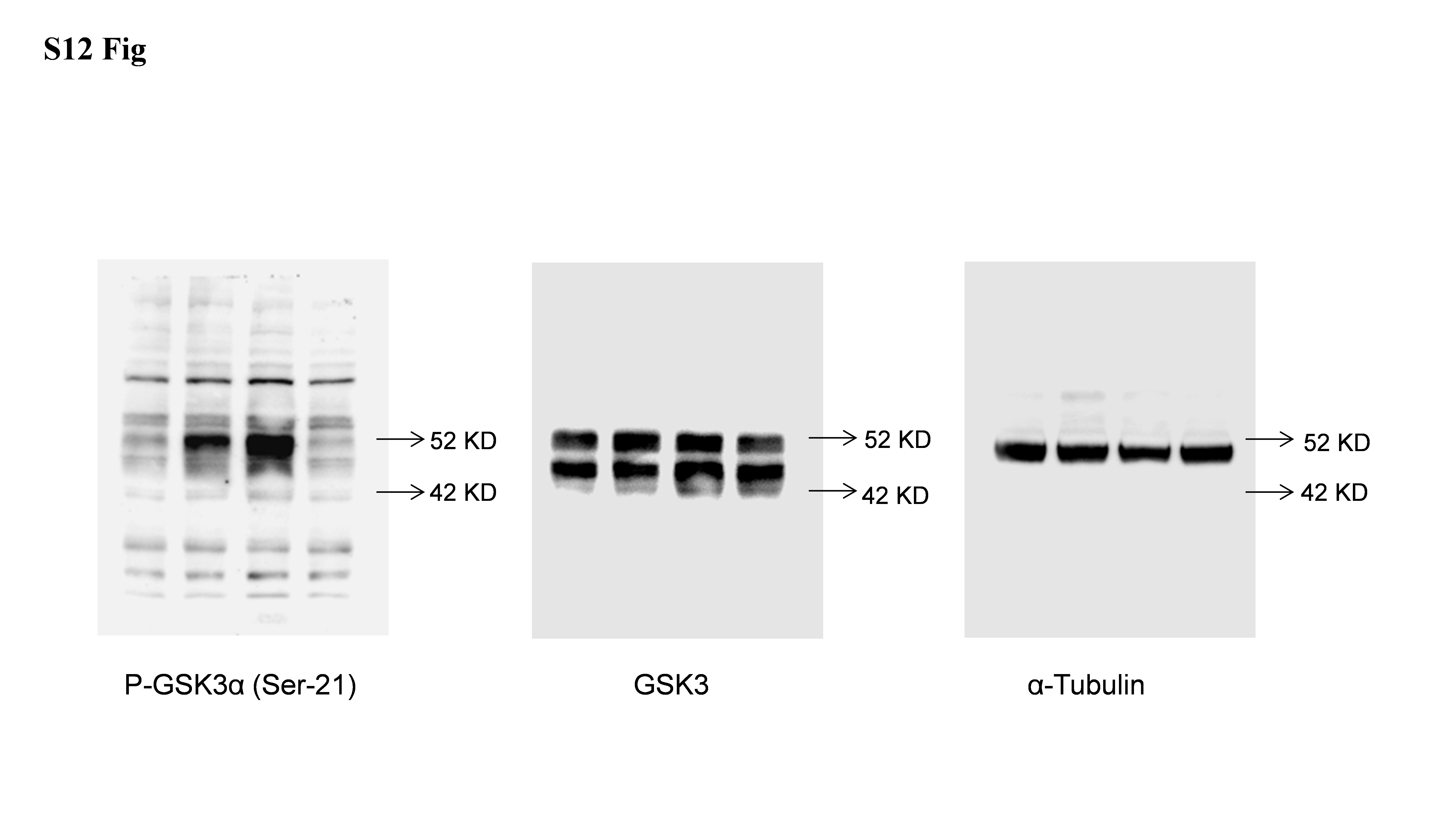

Supplement: S12 Fig — (TIF) [file pone.0224162.s012.tif]

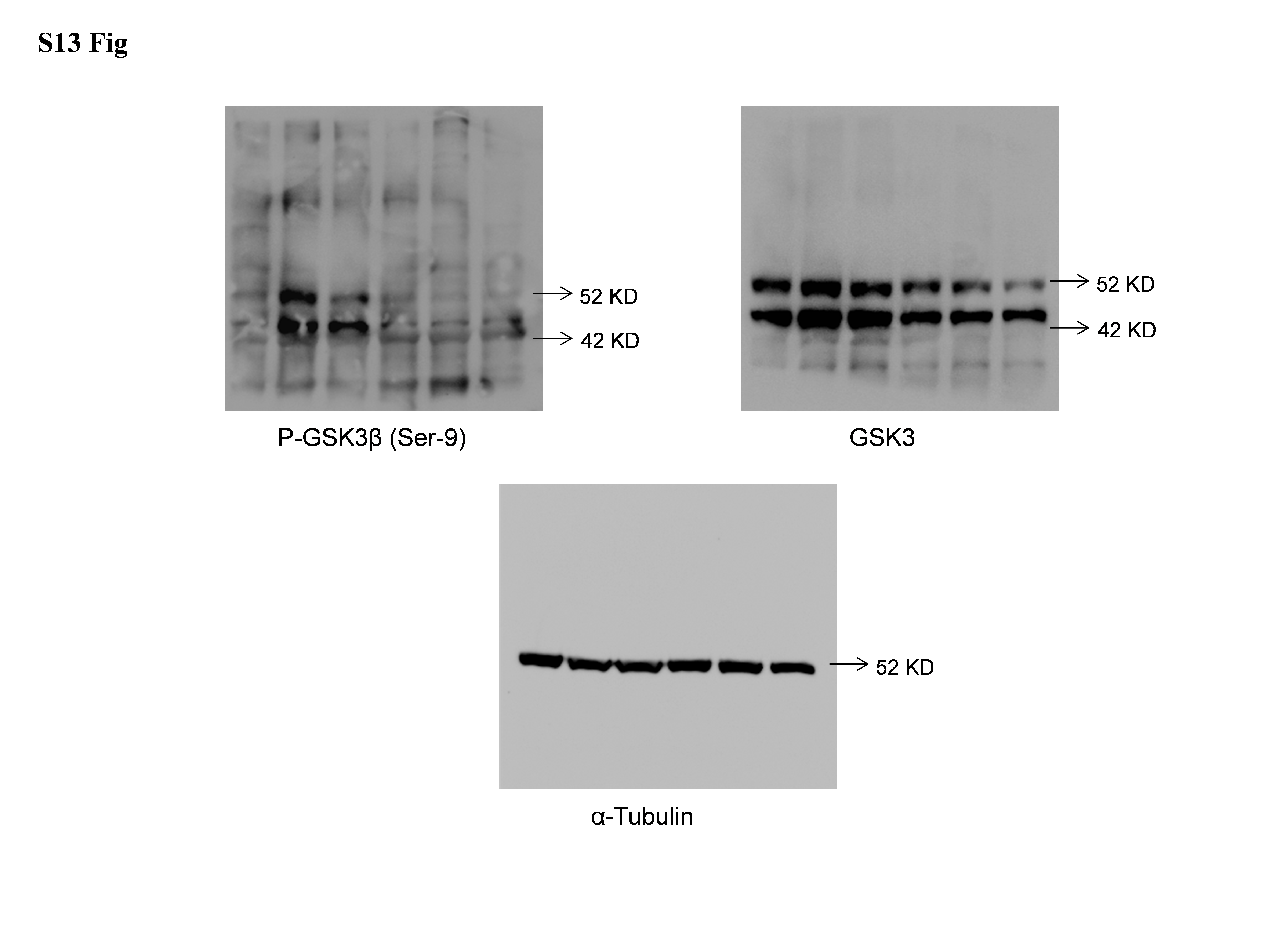

Supplement: S13 Fig — (TIF) [file pone.0224162.s013.tif]

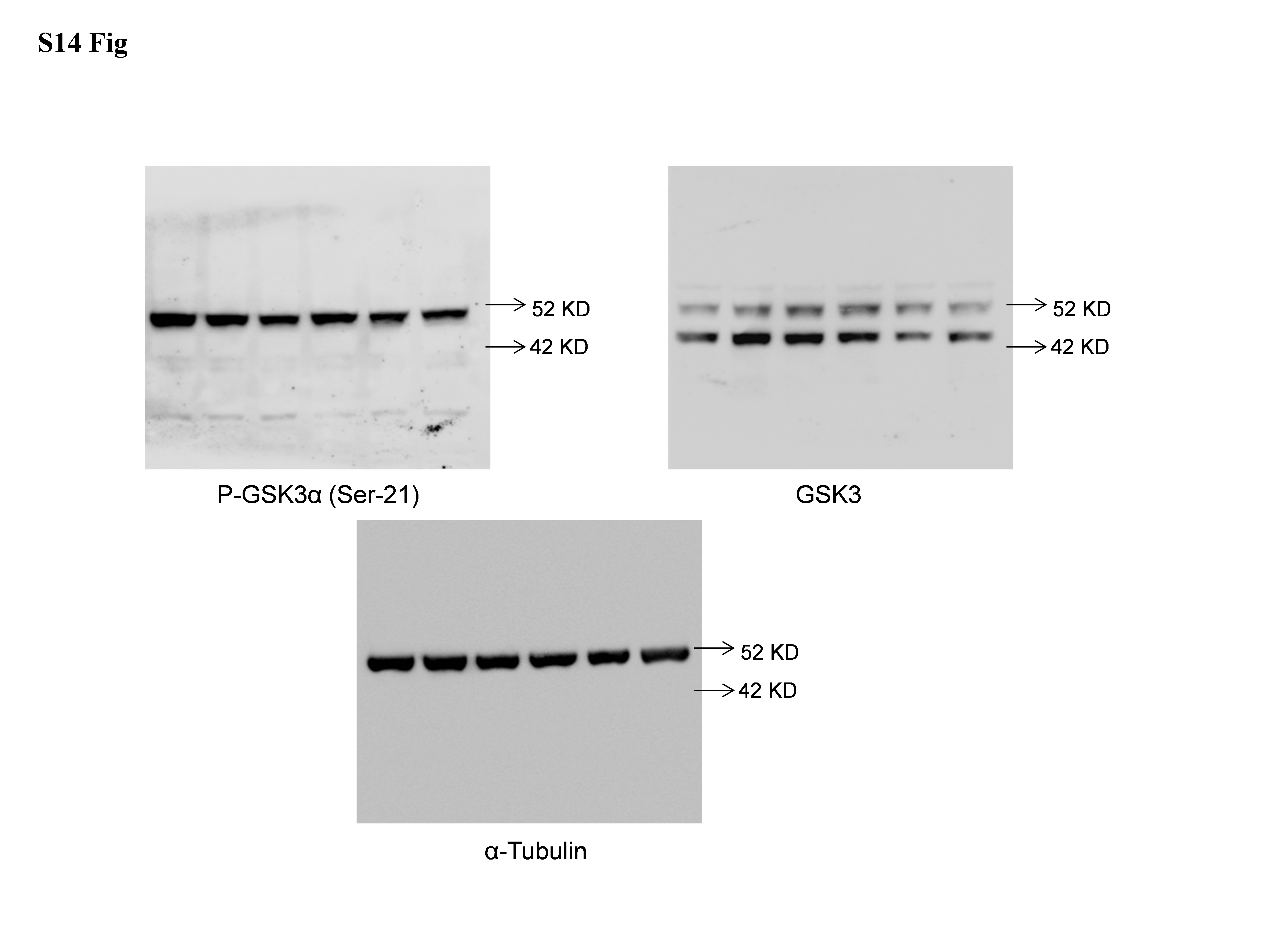

Supplement: S14 Fig — (TIF) [file pone.0224162.s014.tif]

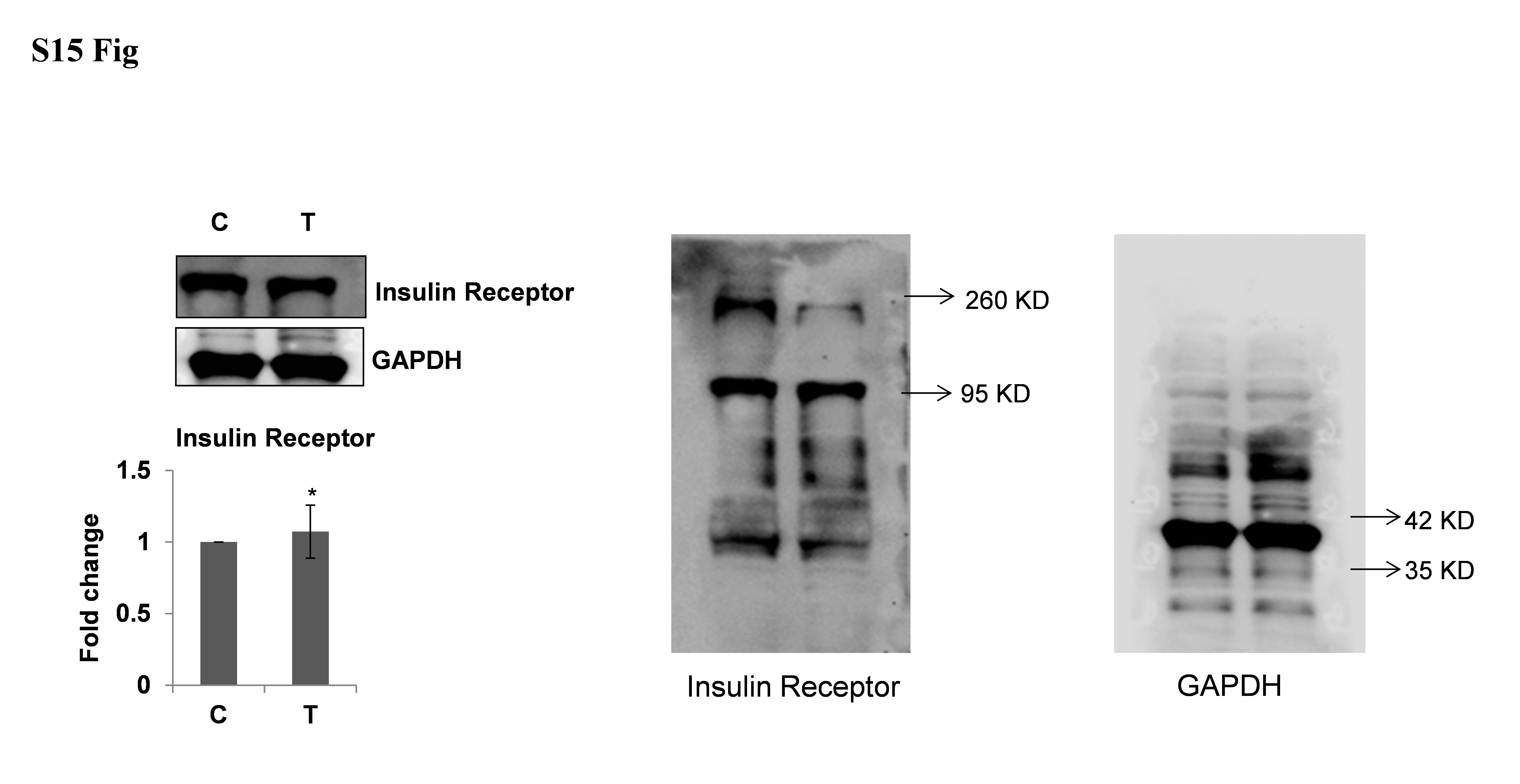

Supplement: S15 Fig — The data were analyzed by t-test, data represents mean ±S.D. of 3 independent experiments (n = 3), * = p>0.05. (TIF) [file pone.0224162.s015.tif]

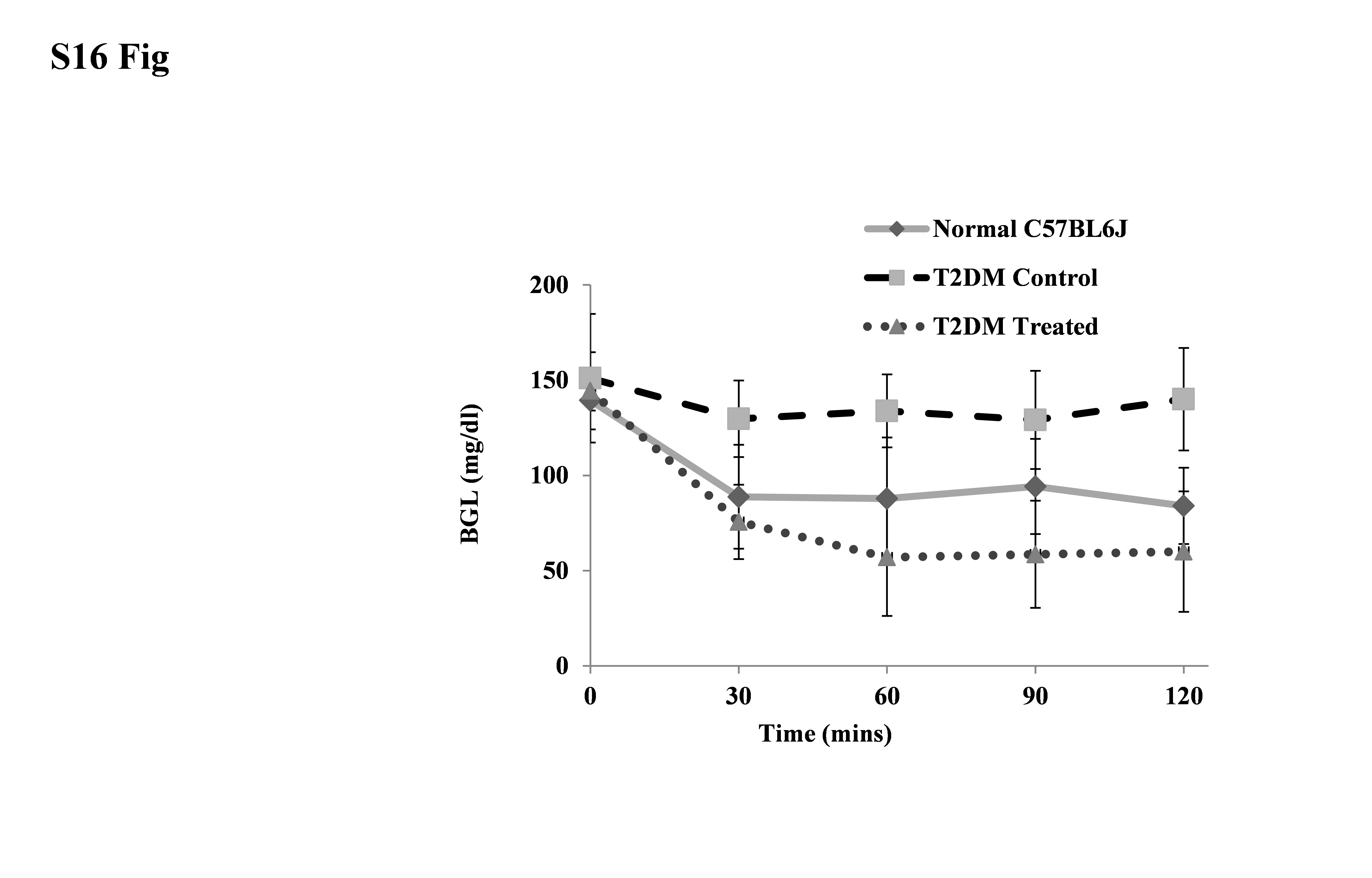

Supplement: S16 Fig — The data were analysed by two-way repeated measures ANOVA test followed by Bonferroni post hoc analysis; data represents mean ±S.D. n = 8, N = 2, p< 0.05, * = no significant difference between the groups. (TIF) [file pone.0224162.s016.tif]
