# Supplementary material for: Testosterone supplementation improves insulin responsiveness in HFD fed male T2DM mice and potentiates insulin signaling in the skeletal muscle and C2C12 myocyte cell line
Source: PLoS One. 2019 Nov 6;14(11):e0224162. doi: 10.1371/journal.pone.0224162 (PMC6834245; doi:10.1371/journal.pone.0224162)
Supplement: S1 Table — N = Normal Chow fed 46 weeks old C57BL6J male mice; C = HFD fed age matched C57BL6J male mice; T = HFD fed age matched C57BL6J male mice supplemented with testosterone. n = 10, p<0.005. # = p>0.1 as compared to C. (DOCX) [file pone.0224162.s017.docx]

**S1 Table**

| **Analyte (pg/ml)**  Mean (±S.D.) | **N** | **C** | **T** |
| --- | --- | --- | --- |
| Insulin | 394.735 (±113.16) | 1315.71 (±163.81) | 1282.06(±141.34) ^#^ |
| Glucagon | 127.95(±39.66) | 537.50(±46.34) | 556.09(±45.79) ^#^ |
| C-Peptide | 421.97 (±153.42 | 2836.03(±422.23) | 2499.67(±421.97) ^#^ |
| IL-6 | 321.89(±148.55) | 1320.52(±256.22) | 1368.03(±187.10) ^#^ |
| MCP-1 | 264.00(±120.06) | 2900.20(±296.17) | 2889.76(±410.39) ^#^ |
| TNF-α | 100.94(±65.55) | 1040.21(±95.02) | 1044.86(±122.96) ^#^ |
| Leptin | 448.85(±413.39) | 4432.20(±920.41) | 4270.71(±772.27) ^#^ |
| PYY | 135.73(±79.75) | 414.08(±52.89) | 447.78(±63.36) ^#^ |
| Resistin | 1197.25(±607.72) | 3675.47(±369.62) | 3782.22(±129.65) ^#^ |
| GIP | 82.78(±34.95) | 367.43(±161.35) | 327.72(±116.03) ^#^ |
