# Supplementary material for: Testosterone supplementation improves insulin responsiveness in HFD fed male T2DM mice and potentiates insulin signaling in the skeletal muscle and C2C12 myocyte cell line
Source: PLoS One. 2019 Nov 6;14(11):e0224162. doi: 10.1371/journal.pone.0224162 (PMC6834245; doi:10.1371/journal.pone.0224162)
Supplement: S2 Table — Blank boxes in table indicate no change in expression level as compared to N; F.C. = Fold Change. (DOCX) [file pone.0224162.s018.docx]

**S2 Table**

| **KINASAE GENE** | **T/N (F.C.)** | **C/N (F.C.)** | **DEFINITION** | **Difference between T and C** |
| --- | --- | --- | --- | --- |
| Map2k6 | -2.430 |  | Mus musculus mitogen activated protein kinase kinase 6 (Map2k6), mRNA. | -2.430 |
| Wnt4 | -2.105 |  | Mus musculus wingless-related MMTV integration site 4 (Wnt4), mRNA. | -2.105 |
| Dapk2 | -1.973 |  | Mus musculus death-associated kinase 2 (Dapk2), mRNA. | -1.973 |
| Styx | 1.820 | 3.789 | Mus musculus serine/threonine/tyrosine interaction protein (Styx), mRNA. | -1.969 |
| Fn3k | -1.797 |  | Mus musculus fructosamine 3 kinase (Fn3k), transcript variant 2, mRNA. | -1.797 |
| Aurka | -1.736 |  | Mus musculus aurora kinase A (Aurka), mRNA. | -1.736 |
| Pdk2 | -1.682 |  | Mus musculus pyruvate dehydrogenase kinase, isoenzyme 2 (Pdk2), mRNA. | -1.682 |
| Dusp10 | -1.642 |  | Mus musculus dual specificity phosphatase 10 (Dusp10), mRNA. | -1.642 |
| Adk | -1.536 |  | Mus musculus adenosine kinase (Adk), mRNA. | -1.536 |
| Ppm1l | -1.523 |  | Mus musculus protein phosphatase 1 (formerly 2C)-like (Ppm1l), mRNA. | -1.523 |
| Srpk3 | -1.472 |  | Mus musculus serine/arginine-rich protein specific kinase 3 (Srpk3), mRNA. | -1.472 |
| Map3k3 | 3.074 | 4.248 | Mus musculus mitogen-activated protein kinase kinase kinase 3 (Map3k3), mRNA. | -1.174 |
| Dusp8 | 1.833 | 3.006 | Mus musculus dual specificity phosphatase 8 (Dusp8), mRNA. | -1.172 |
| Mapk1 |  | 1.161 |  | -1.161 |
| Prkcbp1 | 3.281 | 4.120 | Mus musculus protein kinase C binding protein 1 (Prkcbp1), mRNA. | -0.839 |
| Gsk3b | 1.836 | 2.441 | Mus musculus glycogen synthase kinase 3 beta (Gsk3b), mRNA. | -0.605 |
| Trib2 | -2.104 | -1.648 |  | -0.455 |
| Ipmk | -1.899 | -1.561 | Mus musculus inositol polyphosphate multikinase (Ipmk), mRNA. | -0.338 |
| Pip5k1a | 4.344 | 4.483 | Mus musculus phosphatidylinositol-4-phosphate 5-kinase, type 1 alpha (Pip5k1a), mRNA. | -0.139 |
| Hipk2 | 1.803 | 1.939 | Mus musculus homeodomain interacting protein kinase 2 (Hipk2), mRNA. | -0.136 |
| Csnk1e | 1.575 | 1.671 | Mus musculus casein kinase 1, epsilon (Csnk1e), mRNA. | -0.096 |
| Pfkm | 0.969 | 1.005 | Mus musculus phosphofructokinase, muscle (Pfkm), mRNA. | -0.036 |
| Prkacb | 1.500 | 1.489 | Mus musculus protein kinase, cAMP dependent, catalytic, beta (Prkacb), mRNA. | 0.012 |
| Spag9 | 1.385 | 1.273 | Mus musculus sperm associated antigen 9 (Spag9), transcript variant 4, mRNA. | 0.112 |
| Mast2 | -1.418 | -1.563 | Mus musculus microtubule associated serine/threonine kinase 2 (Mast2), transcript variant 2, mRNA. | 0.146 |
| Stk39 | -1.655 | -1.867 | Mus musculus serine/threonine kinase 39, STE20/SPS1 homolog (yeast) (Stk39), mRNA. | 0.212 |
| Prkcd | 1.974 | 1.615 | Mus musculus protein kinase C, delta (Prkcd), mRNA. | 0.359 |
| Nuak1 | 2.004 | 1.613 | Mus musculus NUAK family, SNF1-like kinase, 1 (Nuak1), mRNA. | 0.392 |
| Map3k14 | -1.631 | -2.298 | Mus musculus mitogen-activated protein kinase kinase kinase 14 (Map3k14), mRNA. | 0.668 |
| Vldlr | 2.252 | 1.557 | Mus musculus very low density lipoprotein receptor (Vldlr), mRNA. | 0.695 |
| Pik3r1 | 3.109 | 1.729 | Mus musculus phosphatidylinositol 3-kinase, regulatory subunit, polypeptide 1 (p85 alpha) (Pik3r1), transcript variant 2, mRNA. | 1.381 |
| S3-12 | 1.443 |  | Mus musculus plasma membrane associated protein, S3-12 (S3-12), mRNA. | 1.443 |
| Sgk1 | 1.466 |  | Mus musculus serum/glucocorticoid regulated kinase 1 (Sgk1), mRNA. | 1.466 |
| Errfi1 | 1.544 |  | Mus musculus ERBB receptor feedback inhibitor 1 (Errfi1), mRNA. | 1.544 |
| Mknk2 | 1.569 |  | Mus musculus MAP kinase-interacting serine/threonine kinase 2 (Mknk2), mRNA. | 1.569 |
| Stat3 | 3.569 | 1.999 | Mus musculus signal transducer and activator of transcription 3 (Stat3), transcript variant 1, mRNA. | 1.570 |
| Rock1 | 1.682 |  | Mus musculus Rho-associated coiled-coil containing protein kinase 1 (Rock1), mRNA. | 1.682 |
| Galk1 | 1.706 |  | Mus musculus galactokinase 1 (Galk1), mRNA. | 1.706 |
| Irs2 | 1.829 |  | Mus musculus insulin receptor substrate 2 (Irs2), mRNA. | 1.829 |
| Flt1 | 1.869 |  | Mus musculus FMS-like tyrosine kinase 1 (Flt1), mRNA. | 1.869 |
| Cdc2l5 | 1.877 |  | Mus musculus cell division cycle 2-like 5 (cholinesterase-related cell division controller) (Cdc2l5), mRNA. | 1.877 |
| Fabp4 | 1.989 |  | Mus musculus fatty acid binding protein 4, adipocyte (Fabp4), mRNA. | 1.989 |
| Map3k8 | 2.066 |  | Mus musculus mitogen-activated protein kinase kinase kinase 8 (Map3k8), mRNA. | 2.066 |
| Mapk6 | 2.072 |  | Mus musculus mitogen-activated protein kinase 6 (Mapk6), transcript variant 2, mRNA. | 2.072 |
| Prkcb | 4.487 | 2.240 | Mus musculus protein kinase C, beta (Prkcb), mRNA. | 2.247 |
| Ar | 2.413 |  | Mus musculus androgen receptor (Ar), mRNA. | 2.413 |
| Musk | 2.681 |  | Mus musculus muscle, skeletal, receptor tyrosine kinase (Musk), transcript variant 3, mRNA. | 2.681 |
| Pdk4 | 1.527 | -1.504 | Mus musculus pyruvate dehydrogenase kinase, isoenzyme 4 (Pdk4), mRNA. | 3.031 |
| Cdkn1a | 4.364 |  | Mus musculus cyclin-dependent kinase inhibitor 1A (P21) (Cdkn1a), mRNA. | 4.364 |
| Gck | 3.425 | -2.452 | Mus musculus glucokinase (Gck), mRNA. | 5.876 |
