# Supplementary material for: Testosterone supplementation improves insulin responsiveness in HFD fed male T2DM mice and potentiates insulin signaling in the skeletal muscle and C2C12 myocyte cell line
Source: PLoS One. 2019 Nov 6;14(11):e0224162. doi: 10.1371/journal.pone.0224162 (PMC6834245; doi:10.1371/journal.pone.0224162)
Supplement: S3 Table — Blank boxes in table indicate no change in expression level as compared to N; F.C. = Fold Change. (DOCX) [file pone.0224162.s019.docx]

**S3 Table**

| \| **GENE** \| **T/N (F.C.)** \| **C/N (F.C.)** \| **DEFINITION** \| **Difference between T and C** \| \| --- \| --- \| --- \| --- \| --- \| \| Hspa1a \| -20.575 \| -20.590 \| Mus musculus heat shock protein 1A (Hspa1a), mRNA. \| 0.015 \| \| Hps5 \| -6.464 \|  \| Mus musculus Hermansky-Pudlak syndrome 5 homolog (human) (Hps5), transcript variant 2, mRNA. \| -6.464 \| \| Cyp4f39 \| -6.409 \|  \| Mus musculus cytochrome P450, family 4, subfamily f, polypeptide 39 (Cyp4f39), mRNA. \| -6.409 \| \| V1rd6 \| -6.247 \|  \| Mus musculus vomeronasal 1 receptor, D6 (V1rd6), mRNA. \| -6.247 \| \| Il12a \| -5.706 \|  \| Mus musculus interleukin 12a (Il12a), mRNA. \| -5.706 \| \| Arc \| -5.214 \|  \| Mus musculus activity regulated cytoskeletal-associated protein (Arc), mRNA. \| -5.214 \| \| Ak3l1 \| -4.645 \|  \| Mus musculus adenylate kinase 3-like 1 (Ak3l1), nuclear gene encoding mitochondrial protein, mRNA. \| -4.645 \| \| Padi2 \| -4.311 \|  \| Mus musculus peptidyl arginine deiminase, type II (Padi2), mRNA. \| -4.311 \| \| Atp1b2 \| -3.901 \|  \| Mus musculus ATPase, Na+/K+ transporting, beta 2 polypeptide (Atp1b2), mRNA. \| -3.901 \| \| Smad3 \| -3.751 \|  \| Mus musculus MAD homolog 3 (Drosophila) (Smad3), mRNA. \| -3.751 \| \| Chrne \| -3.714 \|  \| Mus musculus cholinergic receptor, nicotinic, epsilon polypeptide (Chrne), mRNA. \| -3.714 \| \| Slc23a3 \| -3.622 \|  \| Mus musculus solute carrier family 23 (nucleobase transporters), member 3 (Slc23a3), mRNA. \| -3.622 \| \| Cacnb1 \| -3.327 \|  \| Mus musculus calcium channel, voltage-dependent, beta 1 subunit (Cacnb1), transcript variant variant 2, mRNA. \| -3.327 \| \| Kcnb1 \| -3.265 \|  \| Mus musculus potassium voltage gated channel, Shab-related subfamily, member 1 (Kcnb1), mRNA. \| -3.265 \| \| 2610528E23Rik \| -3.203 \|  \| Mus musculus RIKEN cDNA 2610528E23 gene (2610528E23Rik), mRNA. \| -3.203 \| \| Osgin1 \| -3.078 \| -2.771 \| Mus musculus oxidative stress induced growth inhibitor 1 (Osgin1), mRNA. \| -0.307 \| \| Ctnna3 \| -3.016 \|  \| Mus musculus catenin (cadherin associated protein), alpha 3 (Ctnna3), mRNA. \| -3.016 \| \| Ddah1 \| -2.953 \|  \|  \| -2.953 \| \| P2ry1 \| -2.808 \|  \| Mus musculus purinergic receptor P2Y, G-protein coupled 1 (P2ry1), mRNA. \| -2.808 \| \| Serpinh1 \| -2.754 \|  \| Mus musculus serine (or cysteine) peptidase inhibitor, clade H, member 1 (Serpinh1), mRNA. \| -2.754 \| \| Lrrn4cl \| -2.748 \|  \| Mus musculus LRRN4 C-terminal like (Lrrn4cl), mRNA. \| -2.748 \| \| Rpap3 \| -2.746 \|  \| Mus musculus RNA polymerase II associated protein 3 (Rpap3), mRNA. \| -2.746 \| \| Itgb1bp2 \| -2.740 \|  \| Mus musculus integrin beta 1 binding protein 2 (Itgb1bp2), mRNA. \| -2.740 \| \| A130092J06Rik \| -2.675 \|  \| Mus musculus RIKEN cDNA A130092J06 gene (A130092J06Rik), mRNA. \| -2.675 \| \| Ccl11 \| -2.665 \|  \| Mus musculus small chemokine (C-C motif) ligand 11 (Ccl11), mRNA. \| -2.665 \| \| Mcpt4 \| -2.617 \|  \| Mus musculus mast cell protease 4 (Mcpt4), mRNA. \| -2.617 \| \| Hhatl \| -2.600 \|  \| Mus musculus hedgehog acyltransferase-like (Hhatl), mRNA. \| -2.600 \| \| 9330129D05Rik \| -2.579 \|  \|  \| -2.579 \| \| Gm889 \| -2.530 \|  \| Mus musculus gene model 889, (NCBI) (Gm889), mRNA. \| -2.530 \| \| Igfbp5 \| -2.519 \|  \| Mus musculus insulin-like growth factor binding protein 5 (Igfbp5), mRNA. \| -2.519 \| \| Gsta4 \| -2.504 \|  \| Mus musculus GLUTathione S-transferase, alpha 4 (Gsta4), mRNA. \| -2.504 \| \| Retnla \| -2.503 \|  \| Mus musculus resistin like alpha (Retnla), mRNA. \| -2.503 \| \| Tnfaip2 \| -2.464 \|  \| Mus musculus tumor necrosis factor, alpha-induced protein 2 (Tnfaip2), mRNA. \| -2.464 \| \| Gpd1 \| -2.443 \|  \| Mus musculus glycerol-3-phosphate dehydrogenase 1 (soluble) (Gpd1), mRNA. \| -2.443 \| \| Ppapdc3 \| -2.415 \|  \| Mus musculus phosphatidic acid phosphatase type 2 domain containing 3 (Ppapdc3), mRNA. \| -2.415 \| \| Vamp1 \| -2.377 \|  \|  \| -2.377 \| \| Agpat5 \| -2.367 \|  \| Mus musculus 1-acylglycerol-3-phosphate O-acyltransferase 5 (lysophosphatidic acid acyltransferase, epsilon) (Agpat5), mRNA. \| -2.367 \| \| Cma1 \| -2.330 \|  \| Mus musculus chymase 1, mast cell (Cma1), mRNA. \| -2.330 \| \| Per2 \| -2.321 \|  \| Mus musculus period homolog 2 (Drosophila) (Per2), mRNA. \| -2.321 \| \| Cish \| -2.282 \| -2.942 \| Mus musculus cytokine inducible SH2-containing protein (Cish), mRNA. \| 0.661 \| \| 1110048D14Rik \| -2.256 \|  \|  \| -2.256 \| \| 3010026O09Rik \| -2.250 \|  \| Mus musculus RIKEN cDNA 3010026O09 gene (3010026O09Rik), mRNA. \| -2.250 \| \| Ufsp1 \| -2.247 \|  \| Mus musculus UFM1-specific peptidase 1 (Ufsp1), mRNA. \| -2.247 \| \| Lrrfip1 \| -2.232 \|  \| Mus musculus leucine rich repeat (in FLII) interacting protein 1 (Lrrfip1), mRNA. \| -2.232 \| \| Mrpl35 \| -2.226 \|  \| Mus musculus mitochondrial ribosomal protein L35 (Mrpl35), nuclear gene encoding mitochondrial protein, mRNA. \| -2.226 \| \| Lum \| -2.222 \|  \| Mus musculus lumican (Lum), mRNA. \| -2.222 \| \| Rxrg \| -2.214 \|  \| Mus musculus retinoid X receptor gamma (Rxrg), mRNA. \| -2.214 \| \| Avil \| -2.209 \|  \| Mus musculus advillin (Avil), mRNA. \| -2.209 \| \| Lman2l \| -2.200 \|  \| Mus musculus lectin, mannose-binding 2-like (Lman2l), mRNA. \| -2.200 \| \| 2310045A20Rik \| -2.186 \|  \| Mus musculus RIKEN cDNA 2310045A20 gene (2310045A20Rik), mRNA. \| -2.186 \| \| Tcea3 \| -2.176 \|  \| Mus musculus transcription elongation factor A (SII), 3 (Tcea3), mRNA. \| -2.176 \| \| LOC100046690 \| -2.168 \|  \| PREDICTED: Mus musculus hypothetical protein LOC100046690 (LOC100046690), mRNA. \| -2.168 \| \| Tuba8 \| -2.155 \|  \| Mus musculus tubulin, alpha 8 (Tuba8), mRNA. \| -2.155 \| \| Pxmp2 \| -2.142 \|  \| Mus musculus peroxisomal membrane protein 2 (Pxmp2), mRNA. \| -2.142 \| \| Tmem117 \| -2.124 \|  \| Mus musculus transmembrane protein 117 (Tmem117), mRNA. \| -2.124 \| \| Tiam1 \| -2.119 \|  \| Mus musculus T-cell lymphoma invasion and metastasis 1 (Tiam1), mRNA. \| -2.119 \| \| EG435391 \| -2.109 \|  \| Mus musculus predicted gene, EG435391 (EG435391), mRNA. \| -2.109 \| \| Nkrf \| -2.098 \| -2.610 \| Mus musculus NF-kappaB repressing factor (Nkrf), mRNA. \| 0.512 \| \| Gadd45a \| -2.082 \|  \| Mus musculus growth arrest and DNA-damage-inducible 45 alpha (Gadd45a), mRNA. \| -2.082 \| \| Myo18a \| -2.063 \|  \| Mus musculus myosin XVIIIa (Myo18a), mRNA. \| -2.063 \| \| LOC100046120 \| -2.059 \|  \| PREDICTED: Mus musculus similar to clusterin (LOC100046120), mRNA. \| -2.059 \| \| Hemk1 \| -2.029 \|  \| Mus musculus HemK methyltransferase family member 1 (Hemk1), mRNA. \| -2.029 \| \| Sypl2 \| -2.012 \|  \| Mus musculus synaptophysin-like 2 (Sypl2), mRNA. \| -2.012 \| \| Ahsa2 \| -1.978 \|  \| Mus musculus AHA1, activator of heat shock protein ATPase homolog 2 (yeast) (Ahsa2), mRNA. \| -1.978 \| \| 2310007A19Rik \| -1.952 \|  \| Mus musculus RIKEN cDNA 2310007A19 gene (2310007A19Rik), mRNA. \| -1.952 \| \| Decr1 \| -1.940 \|  \| Mus musculus 2,4-dienoyl CoA reductase 1, mitochondrial (Decr1), nuclear gene encoding mitochondrial protein, mRNA. \| -1.940 \| \| Casq1 \| -1.922 \|  \| Mus musculus calsequestrin 1 (Casq1), nuclear gene encoding mitochondrial protein, mRNA. \| -1.922 \| \| Tmc7 \| -1.921 \|  \| Mus musculus transmembrane channel-like gene family 7 (Tmc7), mRNA. \| -1.921 \| \| Col15a1 \| -1.913 \|  \| Mus musculus collagen, type XV, alpha 1 (Col15a1), mRNA. \| -1.913 \| \| Ecsit \| -1.910 \|  \| Mus musculus ECSIT homolog (Drosophila) (Ecsit), mRNA. \| -1.910 \| \| Ipmk \| -1.899 \|  \| Mus musculus inositol polyphosphate multikinase (Ipmk), mRNA. \| -1.899 \| \| Rtn4ip1 \| -1.899 \|  \| Mus musculus reticulon 4 interacting protein 1 (Rtn4ip1), mRNA. \| -1.899 \| \| Dscr1 \| -1.898 \|  \|  \| -1.898 \| \| Ict1 \| -1.871 \|  \| Mus musculus immature colon carcinoma transcript 1 (Ict1), mRNA. \| -1.871 \| \| 6430571L13Rik \| -1.864 \|  \| Mus musculus RIKEN cDNA 6430571L13 gene (6430571L13Rik), mRNA. \| -1.864 \| \| 5430437P03Rik \| -1.857 \|  \| Mus musculus RIKEN cDNA 5430437P03 gene (5430437P03Rik), mRNA. \| -1.857 \| \| Nudt8 \| -1.828 \|  \| Mus musculus nudix (nucleoside diphosphate linked moiety X)-type motif 8 (Nudt8), mRNA. \| -1.828 \| \| Rdm1 \| -1.827 \|  \| Mus musculus RAD52 motif 1 (Rdm1), mRNA. \| -1.827 \| \| Ky \| -1.771 \|  \| Mus musculus kyphoscoliosis peptidase (Ky), mRNA. \| -1.771 \| \| Snurf \| -1.563 \|  \| Mus musculus SNRPN upstream reading frame (Snurf), mRNA. \| -1.563 \| \| Ddx24 \| 0.980 \| -1.065 \| Mus musculus DEAD (Asp-Glu-Ala-Asp) box polypeptide 24 (Ddx24), mRNA. \| 2.046 \| \| Csnk1e \| 1.575 \| 1.671 \| Mus musculus casein kinase 1, epsilon (Csnk1e), mRNA. \| -0.096 \| \| Smad5 \| 1.789 \|  \| Mus musculus MAD homolog 5 (Drosophila) (Smad5), mRNA. \| 1.789 \| \| Ivns1abp \| 1.872 \| 1.280 \| Mus musculus influenza virus NS1A binding protein (Ivns1abp), transcript variant 2, mRNA. \| 0.592 \| \| H2afy \| 1.923 \| 2.121 \| Mus musculus H2A histone family, member Y (H2afy), mRNA. \| -0.198 \| \| Ide \| 1.944 \|  \| Mus musculus insulin degrading enzyme (Ide), mRNA. \| 1.944 \| \| Gm1157 \| 1.955 \|  \| Mus musculus gene model 1157, (NCBI) (Gm1157), mRNA. \| 1.955 \| \| Lgals3 \| 1.965 \| -2.192 \| Mus musculus lectin, galactose binding, soluble 3 (Lgals3), mRNA. \| 4.158 \| \| Slc10a3 \| 2.015 \|  \| Mus musculus solute carrier family 10 (sodium/bile acid cotransporter family), member 3 (Slc10a3), mRNA. \| 2.015 \| \| Setd1b \| 2.035 \| 2.040 \| Mus musculus SET domain containing 1B (Setd1b), mRNA. \| -0.005 \| \| Pabpn1 \| 2.059 \| 2.054 \| Mus musculus poly(A) binding protein, nuclear 1 (Pabpn1), mRNA. \| 0.006 \| \| Zfp131 \| 2.073 \| 1.749 \| Mus musculus zinc finger protein 131 (Zfp131), mRNA. \| 0.324 \| \| Slc1a3 \| 2.099 \| -1.760 \| Mus musculus solute carrier family 1 (glial high affinity GLUTamate transporter), member 3 (Slc1a3), mRNA. \| 3.859 \| \| Rasa1 \| 2.118 \| 2.343 \| Mus musculus RAS p21 protein activator 1 (Rasa1), mRNA. \| -0.226 \| \| Zdhhc21 \| 2.130 \| 3.109 \| Mus musculus zinc finger, DHHC domain containing 21 (Zdhhc21), mRNA. \| -0.979 \| \| Usp13 \| 2.140 \|  \| Mus musculus ubiquitin specific peptidase 13 (isopeptidase T-3) (Usp13), mRNA. \| 2.140 \| \| Arglu1 \| 2.151 \| 1.952 \| Mus musculus arginine and GLUTamate rich 1 (Arglu1), mRNA. \| 0.199 \| \| Aacs \| 2.153 \|  \| Mus musculus acetoacetyl-CoA synthetase (Aacs), mRNA. \| 2.153 \| \| 2510006D16Rik \| 2.180 \| 2.118 \| Mus musculus RIKEN cDNA 2510006D16 gene (2510006D16Rik), mRNA. \| 0.062 \| \| Cxcl9 \| 2.184 \|  \| Mus musculus chemokine (C-X-C motif) ligand 9 (Cxcl9), mRNA. \| 2.184 \| \| Foxp1 \| 2.191 \| 2.118 \| Mus musculus forkhead box P1 (Foxp1), mRNA. \| 0.074 \| \| LOC100047583 \| 2.204 \| 1.527 \| PREDICTED: Mus musculus similar to apolipoprotein D (LOC100047583), mRNA. \| 0.677 \| \| Lox \| 2.209 \|  \| Mus musculus lysyl oxidase (Lox), mRNA. \| 2.209 \| \| Twf2 \| 2.226 \|  \| Mus musculus twinfilin, actin-binding protein, homolog 2 (Drosophila) (Twf2), mRNA. \| 2.226 \| \| Lrg1 \| 2.246 \| -1.646 \| Mus musculus leucine-rich alpha-2-glycoprotein 1 (Lrg1), mRNA. \| 3.892 \| \| Btbd3 \| 2.257 \| 2.043 \|  \| 0.214 \| \| Ffar2 \| 2.271 \|  \| Mus musculus free fatty acid receptor 2 (Ffar2), mRNA. \| 2.271 \| \| Irx3 \| 2.294 \| 1.832 \|  \| 0.462 \| \| EG433923 \| 2.305 \| 1.487 \| Mus musculus predicted gene, EG433923 (EG433923), mRNA. \| 0.817 \| \| Clec2d \| 2.312 \|  \| Mus musculus C-type lectin domain family 2, member d (Clec2d), mRNA. \| 2.312 \| \| Eef2 \| 2.317 \| 3.513 \| Mus musculus eukaryotic translation elongation factor 2 (Eef2), mRNA. \| -1.196 \| \| Zbtb7a \| 2.336 \| 2.726 \| Mus musculus zinc finger and BTB domain containing 7a (Zbtb7a), mRNA. \| -0.390 \| \| D630048P19Rik \| 2.345 \| 3.397 \|  \| -1.051 \| \| Ccl6 \| 2.367 \| -2.192 \|  \| 4.559 \| \| Cygb \| 2.369 \| 1.813 \| Mus musculus cytoglobin (Cygb), mRNA. \| 0.556 \| \| Cd3eap \| 2.371 \| 2.975 \| Mus musculus CD3E antigen, epsilon polypeptide associated protein (Cd3eap), mRNA. \| -0.603 \| \| Agpat9 \| 2.411 \|  \| Mus musculus 1-acylglycerol-3-phosphate O-acyltransferase 9 (Agpat9), transcript variant 1, mRNA. \| 2.411 \| \| Klhl30 \| 2.412 \|  \| Mus musculus kelch-like 30 (Drosophila) (Klhl30), mRNA. \| 2.412 \| \| Sqstm1 \| 2.419 \| 1.497 \| Mus musculus sequestosome 1 (Sqstm1), mRNA. \| 0.922 \| \| S100a9 \| 2.419 \| 1.901 \| Mus musculus S100 calcium binding protein A9 (calgranulin B) (S100a9), mRNA. \| 0.518 \| \| Opn1mw \| 2.424 \| -1.955 \| Mus musculus opsin 1 (cone pigments), medium-wave-sensitive (color blindness, deutan) (Opn1mw), mRNA. \| 4.379 \| \| Iqgap1 \| 2.445 \| 2.202 \|  \| 0.243 \| \| LOC100046056 \| 2.482 \| 1.693 \| PREDICTED: Mus musculus similar to Pre-B-cell leukemia transcription factor interacting protein 1 (LOC100046056), mRNA. \| 0.789 \| \| Eif4ebp1 \| 2.511 \|  \| Mus musculus eukaryotic translation initiation factor 4E binding protein 1 (Eif4ebp1), mRNA. \| 2.511 \| \| S100a8 \| 2.523 \| 1.987 \| Mus musculus S100 calcium binding protein A8 (calgranulin A) (S100a8), mRNA. \| 0.536 \| \| Inmt \| 2.526 \|  \| Mus musculus indolethylamine N-methyltransferase (Inmt), mRNA. \| 2.526 \| \| Samsn1 \| 2.543 \|  \| Mus musculus SAM domain, SH3 domain and nuclear localization signals, 1 (Samsn1), mRNA. \| 2.543 \| \| Fpr2 \| 2.573 \|  \| Mus musculus formyl peptide receptor 2 (Fpr2), mRNA. \| 2.573 \| \| Atf3 \| 2.643 \|  \| Mus musculus activating transcription factor 3 (Atf3), mRNA. \| 2.643 \| \| Eprs \| 2.652 \| 3.569 \| Mus musculus GLUTamyl-prolyl-tRNA synthetase (Eprs), mRNA. XM_899642 XM_899647 XM_899651 XM_899655 XM_899659 XM_899665 XM_916645 \| -0.917 \| \| LOC100045981 \| 2.656 \| 2.201 \| PREDICTED: Mus musculus similar to synaptotagmin XI (LOC100045981), mRNA. \| 0.455 \| \| BC038156 \| 2.684 \| 4.045 \| Mus musculus cDNA sequence BC038156 (BC038156), mRNA. \| -1.361 \| \| Slc6a12 \| 2.712 \|  \| Mus musculus solute carrier family 6 (neurotransmitter transporter, betaine/GABA), member 12 (Slc6a12), mRNA. \| 2.712 \| \| Nfkbia \| 2.758 \| 1.701 \| Mus musculus nuclear factor of kappa light polypeptide gene enhancer in B-cells inhibitor, alpha (Nfkbia), mRNA. \| 1.057 \| \| Clic5 \| 2.793 \| 2.754 \| Mus musculus chloride intracellular channel 5 (Clic5), mRNA. \| 0.039 \| \| Rnu6 \| 2.794 \| 3.906 \| Mus musculus U6 small nuclear RNA (Rnu6), non-coding RNA. \| -1.112 \| \| Foxn3 \| 2.833 \| 2.765 \| Mus musculus forkhead box N3 (Foxn3), mRNA. \| 0.068 \| \| Gbas \| 2.851 \| 5.748 \| Mus musculus glioblastoma amplified sequence (Gbas), mRNA. \| -2.897 \| \| D16Ertd472e \| 2.888 \| 2.338 \| Mus musculus DNA segment, Chr 16, ERATO Doi 472, expressed (D16Ertd472e), mRNA. \| 0.550 \| \| Fkbp5 \| 2.891 \|  \| Mus musculus FK506 binding protein 5 (Fkbp5), mRNA. \| 2.891 \| \| Odz4 \| 2.897 \| 2.516 \| Mus musculus odd Oz/ten-m homolog 4 (Drosophila) (Odz4), mRNA. \| 0.381 \| \| Dag1 \| 2.898 \| 2.948 \| Mus musculus dystroglycan 1 (Dag1), mRNA. \| -0.049 \| \| Klf13 \| 2.902 \| 2.695 \| Mus musculus Kruppel-like factor 13 (Klf13), mRNA. \| 0.207 \| \| Pdpk1 \| 2.952 \| 2.842 \|  \| 0.110 \| \| 1190002H23Rik \| 3.006 \|  \| Mus musculus RIKEN cDNA 1190002H23 gene (1190002H23Rik), mRNA. \| 3.006 \| \| Mmp14 \| 3.010 \| 2.281 \| Mus musculus matrix metallopeptidase 14 (membrane-inserted) (Mmp14), mRNA. \| 0.729 \| \| Mustn1 \| 3.025 \|  \| Mus musculus musculoskeletal, embryonic nuclear protein 1 (Mustn1), mRNA. \| 3.025 \| \| Gpc3 \| 3.030 \| 5.147 \| Mus musculus glypican 3 (Gpc3), mRNA. \| -2.117 \| \| Cd47 \| 3.043 \| 2.503 \| Mus musculus CD47 antigen (Rh-related antigen, integrin-associated signal transducer) (Cd47), mRNA. \| 0.540 \| \| Mt2 \| 3.066 \|  \| Mus musculus metallothionein 2 (Mt2), mRNA. \| 3.066 \| \| Map3k3 \| 3.074 \| 4.248 \| Mus musculus mitogen-activated protein kinase kinase kinase 3 (Map3k3), mRNA. \| -1.174 \| \| Map3k3 \| 3.074 \| 4.248 \| Mus musculus mitogen-activated protein kinase kinase kinase 3 (Map3k3), mRNA. \| -1.174 \| \| Pik3r1 \| 3.109 \| 1.729 \| Mus musculus phosphatidylinositol 3-kinase, regulatory subunit, polypeptide 1 (p85 alpha) (Pik3r1), transcript variant 2, mRNA. \| 1.381 \| \| Acss1 \| 3.114 \|  \| Mus musculus acyl-CoA synthetase short-chain family member 1 (Acss1), nuclear gene encoding mitochondrial protein, mRNA. \| 3.114 \| \| Bcl9l \| 3.118 \| 3.909 \| Mus musculus B-cell CLL/lymphoma 9-like (Bcl9l), mRNA. \| -0.791 \| \| Napb \| 3.125 \| 2.730 \| Mus musculus N-ethylmaleimide sensitive fusion protein attachment protein beta (Napb), mRNA. \| 0.396 \| \| Zfp292 \| 3.141 \| 3.436 \| PREDICTED: Mus musculus zinc finger protein 292, transcript variant 4 (Zfp292), mRNA. \| -0.295 \| \| Sln \| 3.156 \|  \| Mus musculus sarcolipin (Sln), mRNA. \| 3.156 \| \| Ccnd2 \| 3.191 \| 3.280 \| Mus musculus cyclin D2 (Ccnd2), mRNA. \| -0.089 \| \| Klhl38 \| 3.325 \|  \| Mus musculus kelch-like 38 (Drosophila) (Klhl38), mRNA. \| 3.325 \| \| Wisp2 \| 3.333 \|  \| Mus musculus WNT1 inducible signaling pathway protein 2 (Wisp2), mRNA. \| 3.333 \| \| Gsn \| 3.373 \| 6.158 \| Mus musculus gelsolin (Gsn), mRNA. \| -2.785 \| \| Trp53 \| 3.378 \| 3.845 \| Mus musculus transformation related protein 53 (Trp53), mRNA. \| -0.467 \| \| Cacna2d1 \| 3.414 \| 6.190 \| Mus musculus calcium channel, voltage-dependent, alpha2/delta subunit 1 (Cacna2d1), mRNA. \| -2.776 \| \| Gck \| 3.425 \| -2.452 \| Mus musculus glucokinase (Gck), mRNA. \| 5.876 \| \| Fcgr4 \| 3.441 \|  \| Mus musculus Fc receptor, IgG, low affinity IV (Fcgr4), mRNA. \| 3.441 \| \| Csrp3 \| 3.462 \|  \| Mus musculus cysteine and glycine-rich protein 3 (Csrp3), mRNA. \| 3.462 \| \| Hip1 \| 3.516 \| 5.295 \| Mus musculus huntingtin interacting protein 1 (Hip1), mRNA. \| -1.779 \| \| Appbp2 \| 3.522 \| 2.443 \|  \| 1.078 \| \| Stat3 \| 3.569 \| 1.999 \| Mus musculus signal transducer and activator of transcription 3 (Stat3), transcript variant 1, mRNA. \| 1.570 \| \| Figf \| 3.633 \|  \| Mus musculus c-fos induced growth factor (Figf), mRNA. \| 3.633 \| \| Las1l \| 3.636 \| 4.075 \| Mus musculus LAS1-like (S. cerevisiae) (Las1l), mRNA. \| -0.438 \| \| Trim63 \| 3.640 \|  \| Mus musculus tripartite motif-containing 63 (Trim63), mRNA. \| 3.640 \| \| Hist1h2ao \| 3.682 \| 4.648 \| Mus musculus histone cluster 1, H2ao (Hist1h2ao), mRNA. \| -0.967 \| \| Cytip \| 3.777 \|  \| Mus musculus cytohesin 1 interacting protein (Cytip), mRNA. \| 3.777 \| \| Ankrd1 \| 3.790 \|  \| Mus musculus ankyrin repeat domain 1 (cardiac muscle) (Ankrd1), mRNA. \| 3.790 \| \| R3hdm1 \| 3.807 \| 5.557 \| Mus musculus R3H domain 1 (binds single-stranded nucleic acids) (R3hdm1), mRNA. \| -1.750 \| \| Dmkn \| 3.833 \|  \| Mus musculus dermokine (Dmkn), transcript variant 2, mRNA. \| 3.833 \| \| Itga7 \| 3.849 \| 4.635 \| Mus musculus integrin alpha 7 (Itga7), mRNA. \| -0.786 \| \| Myd116 \| 3.871 \|  \| Mus musculus myeloid differentiation primary response gene 116 (Myd116), mRNA. \| 3.871 \| \| Rsph1 \| 3.874 \|  \| Mus musculus radial spoke head 1 homolog (Chlamydomonas) (Rsph1), mRNA. \| 3.874 \| \| Agt \| 3.933 \|  \| Mus musculus angiotensinogen (serpin peptidase inhibitor, clade A, member 8) (Agt), mRNA. \| 3.933 \| \| Tnfrsf12a \| 3.968 \|  \| Mus musculus tumor necrosis factor receptor superfamily, member 12a (Tnfrsf12a), mRNA. \| 3.968 \| \| Ppargc1a \| 3.988 \| 2.277 \| Mus musculus peroxisome proliferative activated receptor, gamma, coactivator 1 alpha (Ppargc1a), mRNA. \| 1.712 \| \| Was \| 4.037 \| 2.281 \| Mus musculus Wiskott-Aldrich syndrome homolog (human) (Was), mRNA. \| 1.756 \| \| Samd4 \| 4.099 \| 3.342 \| Mus musculus sterile alpha motif domain containing 4 (Samd4), transcript variant 1, mRNA. \| 0.757 \| \| Vcam1 \| 4.143 \|  \| Mus musculus vascular cell adhesion molecule 1 (Vcam1), mRNA. \| 4.143 \| \| Adap2 \| 4.165 \| 3.121 \| Mus musculus ArfGAP with dual PH domains 2 (Adap2), mRNA. \| 1.044 \| \| Apoc1 \| 4.170 \|  \| Mus musculus apolipoprotein C-I (Apoc1), mRNA. \| 4.170 \| \| Ddit4 \| 4.173 \| 1.582 \| Mus musculus DNA-damage-inducible transcript 4 (Ddit4), mRNA. \| 2.591 \| \| Prkaa2 \| 4.190 \| 7.152 \|  \| -2.962 \| \| Pip5k1a \| 4.344 \| 4.483 \| Mus musculus phosphatidylinositol-4-phosphate 5-kinase, type 1 alpha (Pip5k1a), mRNA. \| -0.139 \| \| Cdkn1a \| 4.364 \|  \| Mus musculus cyclin-dependent kinase inhibitor 1A (P21) (Cdkn1a), mRNA. \| 4.364 \| \| Gna13 \| 4.454 \| 3.732 \|  \| 0.722 \| \| Prkcb \| 4.487 \| 2.240 \| Mus musculus protein kinase C, beta (Prkcb), mRNA. \| 2.247 \| \| Pcyt1a \| 4.771 \| 7.219 \| Mus musculus phosphate cytidylyltransferase 1, choline, alpha isoform (Pcyt1a), mRNA. \| -2.448 \| \| Cpeb3 \| 4.849 \| 10.250 \| Mus musculus cytoplasmic polyadenylation element binding protein 3 (Cpeb3), mRNA. \| -5.402 \| \| Bbx \| 4.903 \| 10.541 \| Mus musculus bobby sox homolog (Drosophila) (Bbx), mRNA. \| -5.638 \| \| Tbc1d20 \| 5.073 \| 5.004 \| Mus musculus TBC1 domain family, member 20 (Tbc1d20), mRNA. \| 0.070 \| \| P2ry13 \| 5.325 \|  \| Mus musculus purinergic receptor P2Y, G-protein coupled 13 (P2ry13), mRNA. \| 5.325 \| \| Gna11 \| 5.563 \|  \|  \| 5.563 \| \| Eif4ebp2 \| 5.807 \| 8.625 \| Mus musculus eukaryotic translation initiation factor 4E binding protein 2 (Eif4ebp2), mRNA. \| -2.817 \| \| Reep3 \| 5.963 \|  \| Mus musculus receptor accessory protein 3 (Reep3), mRNA. \| 5.963 \| \| Prg2 \| 6.199 \| 6.935 \| Mus musculus proteoglycan 2, bone marrow (Prg2), mRNA. \| -0.736 \| \| Mfsd11 \| 6.320 \| 5.570 \| Mus musculus major facilitator superfamily domain containing 11 (Mfsd11), mRNA. \| 0.750 \| \| Selp \| 6.322 \| 1.775 \| Mus musculus selectin, platelet (Selp), mRNA. \| 4.548 \| \| Fbxo32 \| 6.543 \| 1.466 \| Mus musculus F-box protein 32 (Fbxo32), mRNA. \| 5.077 \| \| Fam134b \| 6.954 \|  \| Mus musculus family with sequence similarity 134, member B (Fam134b), transcript variant 2, mRNA. \| 6.954 \| \| Serpina3n \| 7.067 \|  \| Mus musculus serine (or cysteine) peptidase inhibitor, clade A, member 3N (Serpina3n), mRNA. \| 7.067 \| \| Hp \| 7.211 \|  \| Mus musculus haptoglobin (Hp), mRNA. \| 7.211 \| \| Bach1 \| 7.709 \|  \| Mus musculus BTB and CNC homology 1 (Bach1), mRNA. \| 7.709 \| \| Cyp2e1 \| 8.161 \|  \| Mus musculus cytochrome P450, family 2, subfamily e, polypeptide 1 (Cyp2e1), mRNA. \| 8.161 \| \| Klhl9 \| 8.410 \| 9.521 \| Mus musculus kelch-like 9 (Drosophila) (Klhl9), mRNA. \| -1.111 \| \| Sync \| 8.504 \|  \| Mus musculus syncoilin (Sync), mRNA. \| 8.504 \| \| 6430548M08Rik \| 11.255 \| 4.444 \|  \| 6.811 \| \| Hnrpl \| 13.162 \| 11.300 \| Mus musculus heterogeneous nuclear ribonucleoprotein L (Hnrpl), mRNA. \| 1.862 \| \| Pdia4 \| 14.157 \| 8.657 \| Mus musculus protein disulfide isomerase associated 4 (Pdia4), mRNA. \| 5.500 \| \| 2010001M09Rik \| 14.470 \|  \| Mus musculus RIKEN cDNA 2010001M09 gene (2010001M09Rik), mRNA. \| 14.470 \| \| Cxcl13 \| 14.893 \|  \|  \| 14.893 \| \| Lcn2 \| 18.163 \|  \| Mus musculus lipocalin 2 (Lcn2), mRNA. \| 18.163 \| \| Saa3 \| 62.800 \|  \| Mus musculus serum amyloid A 3 (Saa3), mRNA. \| 62.800 \| \| Ppargc1b \|  \| 1.592 \| Mus musculus peroxisome proliferative activated receptor, gamma, coactivator 1 beta (Ppargc1b), mRNA. \| -1.592 \| \| Cebpb \|  \| -1.881 \| Mus musculus CCAAT/enhancer binding protein (C/EBP), beta (Cebpb), mRNA. \| 1.881 \| |  |  |  |  |
| --- | --- | --- | --- | --- | --- | --- | --- | --- | --- | --- | --- | --- | --- | --- | --- | --- | --- | --- | --- | --- | --- | --- | --- | --- | --- | --- | --- | --- | --- | --- | --- | --- | --- | --- | --- | --- | --- | --- | --- | --- | --- | --- | --- | --- | --- | --- | --- | --- | --- | --- | --- | --- | --- | --- | --- | --- | --- | --- | --- | --- | --- | --- | --- | --- | --- | --- | --- | --- | --- | --- | --- | --- | --- | --- | --- | --- | --- | --- | --- | --- | --- | --- | --- | --- | --- | --- | --- | --- | --- | --- | --- | --- | --- | --- | --- | --- | --- | --- | --- | --- | --- | --- | --- | --- | --- | --- | --- | --- | --- | --- | --- | --- | --- | --- | --- | --- | --- | --- | --- | --- | --- | --- | --- | --- | --- | --- | --- | --- | --- | --- | --- | --- | --- | --- | --- | --- | --- | --- | --- | --- | --- | --- | --- | --- | --- | --- | --- | --- | --- | --- | --- | --- | --- | --- | --- | --- | --- | --- | --- | --- | --- | --- | --- | --- | --- | --- | --- | --- | --- | --- | --- | --- | --- | --- | --- | --- | --- | --- | --- | --- | --- | --- | --- | --- | --- | --- | --- | --- | --- | --- | --- | --- | --- | --- | --- | --- | --- | --- | --- | --- | --- | --- | --- | --- | --- | --- | --- | --- | --- | --- | --- | --- | --- | --- | --- | --- | --- | --- | --- | --- | --- | --- | --- | --- | --- | --- | --- | --- | --- | --- | --- | --- | --- | --- | --- | --- | --- | --- | --- | --- | --- | --- | --- | --- | --- | --- | --- | --- | --- | --- | --- | --- | --- | --- | --- | --- | --- | --- | --- | --- | --- | --- | --- | --- | --- | --- | --- | --- | --- | --- | --- | --- | --- | --- | --- | --- | --- | --- | --- | --- | --- | --- | --- | --- | --- | --- | --- | --- | --- | --- | --- | --- | --- | --- | --- | --- | --- | --- | --- | --- | --- | --- | --- | --- | --- | --- | --- | --- | --- | --- | --- | --- | --- | --- | --- | --- | --- | --- | --- | --- | --- | --- | --- | --- | --- | --- | --- | --- | --- | --- | --- | --- | --- | --- | --- | --- | --- | --- | --- | --- | --- | --- | --- | --- | --- | --- | --- | --- | --- | --- | --- | --- | --- | --- | --- | --- | --- | --- | --- | --- | --- | --- | --- | --- | --- | --- | --- | --- | --- | --- | --- | --- | --- | --- | --- | --- | --- | --- | --- | --- | --- | --- | --- | --- | --- | --- | --- | --- | --- | --- | --- | --- | --- | --- | --- | --- | --- | --- | --- | --- | --- | --- | --- | --- | --- | --- | --- | --- | --- | --- | --- | --- | --- | --- | --- | --- | --- | --- | --- | --- | --- | --- | --- | --- | --- | --- | --- | --- | --- | --- | --- | --- | --- | --- | --- | --- | --- | --- | --- | --- | --- | --- | --- | --- | --- | --- | --- | --- | --- | --- | --- | --- | --- | --- | --- | --- | --- | --- | --- | --- | --- | --- | --- | --- | --- | --- | --- | --- | --- | --- | --- | --- | --- | --- | --- | --- | --- | --- | --- | --- | --- | --- | --- | --- | --- | --- | --- | --- | --- | --- | --- | --- | --- | --- | --- | --- | --- | --- | --- | --- | --- | --- | --- | --- | --- | --- | --- | --- | --- | --- | --- | --- | --- | --- | --- | --- | --- | --- | --- | --- | --- | --- | --- | --- | --- | --- | --- | --- | --- | --- | --- | --- | --- | --- | --- | --- | --- | --- | --- | --- | --- | --- | --- | --- | --- | --- | --- | --- | --- | --- | --- | --- | --- | --- | --- | --- | --- | --- | --- | --- | --- | --- | --- | --- | --- | --- | --- | --- | --- | --- | --- | --- | --- | --- | --- | --- | --- | --- | --- | --- | --- | --- | --- | --- | --- | --- | --- | --- | --- | --- | --- | --- | --- | --- | --- | --- | --- | --- | --- | --- | --- | --- | --- | --- | --- | --- | --- | --- | --- | --- | --- | --- | --- | --- | --- | --- | --- | --- | --- | --- | --- | --- | --- | --- | --- | --- | --- | --- | --- | --- | --- | --- | --- | --- | --- | --- | --- | --- | --- | --- | --- | --- | --- | --- | --- | --- | --- | --- | --- | --- | --- | --- | --- | --- | --- | --- | --- | --- | --- | --- | --- | --- | --- | --- | --- | --- | --- | --- | --- | --- | --- | --- | --- | --- | --- | --- | --- | --- | --- | --- | --- | --- | --- | --- | --- | --- | --- | --- | --- | --- | --- | --- | --- | --- | --- | --- | --- | --- | --- | --- | --- | --- | --- | --- | --- | --- | --- | --- | --- | --- | --- | --- | --- | --- | --- | --- | --- | --- | --- | --- | --- | --- | --- | --- | --- | --- | --- | --- | --- | --- | --- | --- | --- | --- | --- | --- | --- | --- | --- | --- | --- | --- | --- | --- | --- | --- | --- | --- | --- | --- | --- | --- | --- | --- | --- | --- | --- | --- | --- | --- | --- | --- | --- | --- | --- | --- | --- | --- | --- | --- | --- | --- | --- | --- | --- | --- | --- | --- | --- | --- | --- | --- | --- | --- | --- | --- | --- | --- | --- | --- | --- | --- | --- | --- | --- | --- | --- | --- | --- | --- | --- | --- | --- | --- | --- | --- | --- | --- | --- | --- | --- | --- | --- | --- | --- | --- | --- | --- | --- | --- | --- | --- | --- | --- | --- | --- | --- | --- | --- | --- | --- | --- | --- | --- | --- | --- | --- | --- | --- | --- | --- | --- | --- | --- | --- | --- | --- | --- | --- | --- | --- | --- | --- | --- | --- | --- | --- | --- | --- | --- | --- | --- | --- | --- | --- | --- | --- | --- | --- | --- | --- | --- | --- | --- | --- | --- | --- | --- | --- | --- | --- | --- | --- | --- | --- | --- | --- | --- | --- | --- | --- | --- | --- | --- | --- | --- | --- | --- | --- | --- | --- | --- | --- | --- | --- | --- | --- | --- | --- | --- | --- | --- | --- | --- | --- | --- | --- | --- | --- | --- | --- | --- | --- | --- | --- | --- | --- | --- | --- | --- | --- | --- | --- | --- | --- | --- | --- | --- | --- | --- | --- | --- | --- | --- | --- | --- | --- | --- | --- | --- | --- | --- | --- | --- | --- | --- | --- | --- | --- | --- | --- | --- | --- | --- | --- | --- | --- | --- | --- | --- | --- | --- | --- | --- | --- | --- | --- | --- | --- | --- | --- | --- | --- | --- | --- | --- | --- | --- | --- | --- | --- | --- | --- | --- | --- | --- | --- | --- | --- | --- | --- | --- | --- | --- | --- | --- | --- | --- | --- | --- | --- | --- | --- | --- | --- | --- | --- | --- | --- | --- | --- | --- | --- | --- | --- | --- | --- | --- | --- | --- | --- | --- | --- | --- | --- | --- | --- | --- | --- | --- | --- | --- | --- | --- | --- | --- | --- | --- | --- | --- | --- | --- | --- | --- | --- | --- | --- | --- | --- | --- | --- | --- | --- | --- | --- | --- | --- | --- | --- | --- | --- | --- | --- | --- | --- | --- | --- | --- | --- | --- | --- | --- | --- | --- | --- | --- | --- | --- | --- | --- | --- | --- | --- | --- | --- | --- | --- | --- | --- | --- | --- | --- | --- | --- | --- | --- | --- | --- | --- | --- | --- | --- | --- | --- | --- | --- | --- | --- | --- | --- | --- | --- | --- | --- |
|  |  |  |  |  |
|  |  |  |  |  |
